# Prediction of Medical Disputes Between Health Care Workers and Patients in Terms of Hospital Legal Construction Using Machine Learning Techniques: Externally Validated Cross-Sectional Study

**DOI:** 10.2196/46854

**Published:** 2023-08-17

**Authors:** Min Yi, Yuebin Cao, Lin Wang, Yaowen Gu, Xueqian Zheng, Jiangjun Wang, Wei Chen, Liangyu Wei, Yujin Zhou, Chenyi Shi, Yanlin Cao

**Affiliations:** 1 Institute of Medical Information and Library, Chinese Academy of Medical Sciences and Peking Union Medical College Beijing China; 2 Health Commission of Hunan Province Changsha China; 3 Beijing Municipal Health Commission Beijing China; 4 Chinese Hospital Association Medical Legality Specialized Committee Beijing China; 5 China-Japan Friendship Hospital Beijing China; 6 Beijing Stomatological Hospital, Capital Medical University Beijing China; 7 Beijing Hospital Beijing China

**Keywords:** medical workers, medical disputes, hospital legal construction, machine learning, multicenter analysis

## Abstract

**Background:**

Medical disputes are a global public health issue that is receiving increasing attention. However, studies investigating the relationship between hospital legal construction and medical disputes are scarce. The development of a multicenter model incorporating machine learning (ML) techniques for the individualized prediction of medical disputes would be beneficial for medical workers.

**Objective:**

This study aimed to identify predictors related to medical disputes from the perspective of hospital legal construction and the use of ML techniques to build models for predicting the risk of medical disputes.

**Methods:**

This study enrolled 38,053 medical workers from 130 tertiary hospitals in Hunan province, China. The participants were randomly divided into a training cohort (34,286/38,053, 90.1%) and an internal validation cohort (3767/38,053, 9.9%). Medical workers from 87 tertiary hospitals in Beijing were included in an external validation cohort (26,285/26,285, 100%). This study used logistic regression and 5 ML techniques: decision tree, random forest, support vector machine, gradient boosting decision tree (GBDT), and deep neural network. In total, 12 metrics, including discrimination and calibration, were used for performance evaluation. A scoring system was developed to select the optimal model. Shapley additive explanations was used to generate the importance coefficients for characteristics. To promote the clinical practice of our proposed optimal model, reclassification of patients was performed, and a web-based app for medical dispute prediction was created, which can be easily accessed by the public.

**Results:**

Medical disputes occurred among 46.06% (17,527/38,053) of the medical workers in Hunan province, China. Among the 26 clinical characteristics, multivariate analysis demonstrated that 18 characteristics were significantly associated with medical disputes, and these characteristics were used for ML model development. Among the ML techniques, GBDT was identified as the optimal model, demonstrating the lowest Brier score (0.205), highest area under the receiver operating characteristic curve (0.738, 95% CI 0.722-0.754), and the largest discrimination slope (0.172) and Youden index (1.355). In addition, it achieved the highest metrics score (63 points), followed by deep neural network (46 points) and random forest (45 points), in the internal validation set. In the external validation set, GBDT still performed comparably, achieving the second highest metrics score (52 points). The high-risk group had more than twice the odds of experiencing medical disputes compared with the low-risk group.

**Conclusions:**

We established a prediction model to stratify medical workers into different risk groups for encountering medical disputes. Among the 5 ML models, GBDT demonstrated the optimal comprehensive performance and was used to construct the web-based app. Our proposed model can serve as a useful tool for identifying medical workers at high risk of medical disputes. We believe that preventive strategies should be implemented for the high-risk group.

## Introduction

Medical disputes often arise from differences in the perceptions of treatment outcomes between patients and physicians [1]. This discord between physicians and patients has transformed into a major public health problem, leading to tensions, disputes, and workplace violence [2]. Studies have reported that 33.48% to 76% of medical workers have experienced medical disputes [3] or workplace violence [4] worldwide. The detrimental effects of medical disputes on the physician-patient relationship have become increasingly evident [5], calling for rigorous research and policy interventions.

The identification of predictors for medical disputes is crucial for guiding the implementation of preventive strategies. Previous studies have highlighted that institutional failures in the legal framework, inappropriate internal incentives, patient-physician mistrust, heavy physician workloads, and medical malpractice are risk factors [6-8] contributing to medical disputes. Conversely, being female, implementing positive psychology interventions, and adopting healthy lifestyle management responsibilities are protective factors for medical disputes [9-12]. Previously, we found that hospital legal constructions play an important role in regulating medical disputes from the perspective of hospital administrators [12]. Well-constructed legal frameworks enhance the ability of hospital administrators to prevent and resolve medical disputes.

However, studies investigating the relationship between medical disputes and hospital legal constructions have not fully addressed the role of medical workers. In addition, the development of a prediction model for medical disputes would be beneficial for conducting personalized interventions, as the risk probability of experiencing medical disputes can be accurately evaluated. Nonetheless, no prediction model has been developed for physicians and nurses to evaluate the risk of experiencing medical disputes. Currently, machine learning (ML) is widely used to establish accurate prediction models in medicine [13,14], and it can be used to redefine patient classifications and create reliable risk or diagnostic models using clinical data sets [15,16]. ML techniques provide more accurate diagnostic techniques and personalized patient therapy compared with expert-based or statistical methods [17,18]. This technology is already being successfully applied in real-world apps across various medical professions [17,19-21].

To address these gaps, this study aimed to propose an accurate prediction model based on hospital legal construction to stratify medical staff according to their risk of experiencing medical disputes in the upcoming year. Logistic regression (LR) and 5 ML techniques—decision tree (DT), random forest (RF), support vector machine (SVM), gradient boosting DT (GBDT), and deep neural network (DNN)—were used to train and optimize the models. We hypothesized that these models would be able to identify medical staff who are most likely to experience medical disputes, thereby enabling the early implementation of preventive measures.

## Methods

### Study Design

This study enrolled 38,053 medical staff from 130 tertiary hospitals in Hunan province, China, between July and September 2021 based on a survey that was developed after an extensive literature review and consultations with investigators and senior professors. In addition, between September and October 2022, the survey was officially distributed by the Beijing Health Commission, and we included medical workers from 87 tertiary hospitals in Beijing in the analysis, resulting in a sample size of 26,285. The survey collected data on participants’ basic demographics, occupation, hospital information, the construction status of the hospital rule of law, and their knowledge of medical laws.

**Figure 1:**
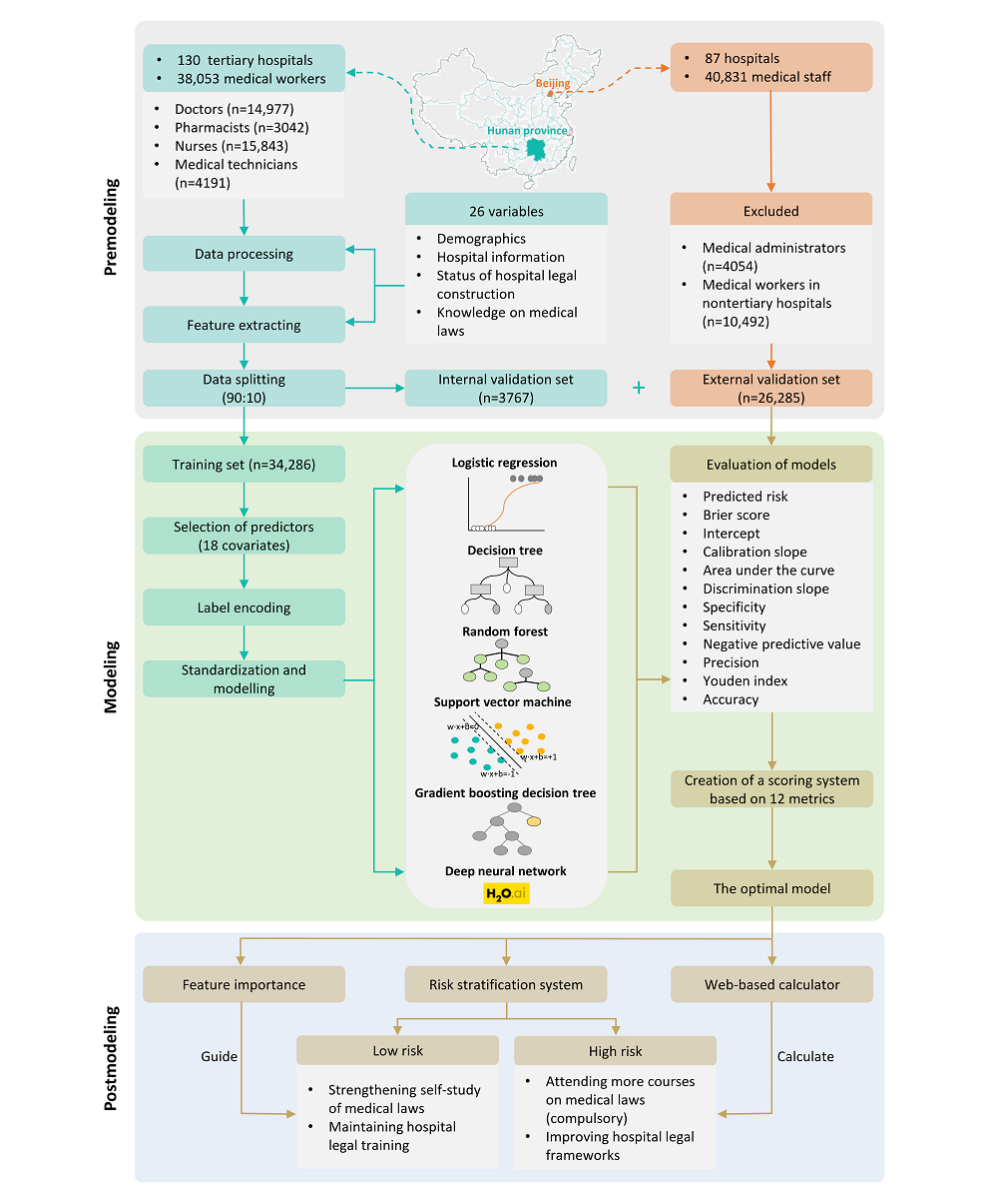
Flowchart outlining the participants’ enrollment and study profile. Participants from the Hunan province were randomly divided into a training set and an internal validation set. The logistic regression model and 5 machine learning models were trained and optimized in the training set, and the internal validation set was used to internally validate the models. Participants from Beijing served as the external validation set. A scoring system was developed after incorporating 21 metrics to assess the prediction performance of the models.

The survey was distributed by the Hunan Provincial Health Commission to the lower-level health administrative departments. A sampling protocol was followed (more details can be found in Multimedia Appendix 1). We included physicians, nurses, pharmacists, and medical technicians working in a tertiary hospital and excluded (1) administrative staff, medical students, and logistics staff; (2) individuals who were reluctant to participate in the survey; and (3) those who were unable to cooperate for other reasons. All enrolled medical workers from Hunan province were randomly split in a 9:1 ratio into a training set (34,286/38,053, 90.1%) and an internal validation set (3767/38,053, 9.9%), and medical workers from Beijing served as the external validation set. The training set was used to train and optimize the models, whereas the internal and external validation sets were used to validate the models. Figure 1 shows the flowchart and protocol design of the study.

### Data Collection

The potential predictors collected in the study can be categorized as follows: (1) basic demographics, including age, sex, occupation, and technical title; (2) hospital information, including hospital type, hospital category, and tertiary hospital level; (3) evaluation of the construction of the hospital rule of law, including the establishment of hospital legal construction, independent rule of law department in the hospital, hospital performance appraisal system including hospital legal construction, rule of law training for new recruits, examination of law popularization among hospital staff, legal training organized by the hospital, publicity of rule of law for hospital staff, publicity of rule of law for patients’ family members, and the construction status of the hospital rule of law; (4) participants’ knowledge on medical laws, including understanding the hospital president responsibility system under the leadership of the hospital party committee, understanding the duty of the hospital law department, understanding the duty of the legal counselor in the hospital, and understanding the contents of hospital charters; and (5) participants’ awareness of medical laws, including the importance of clinical practice in accordance with the law, previous experience of legal issues outside of medical work, willingness to participate in law training organized by the hospital, helpfulness of the hospital’s legal construction to their medical work, participation in legal training organized by hospitals, and the necessity of carrying out legal training in hospitals. All these clinical characteristics were self-reported by participants based on their actual conditions.

In this study, the abovementioned characteristics were clearly defined as follows. Hospital charters refer to the legal frameworks of the hospital, including state laws, administrative regulations, and hospital regulations and rules. The performance appraisal system including hospital legal construction indicates that hospital legal construction was considered an important item in the performance appraisal system. More detailed information on the definition of each characteristic is provided in Multimedia Appendix 1.

### Development and Validation of Models

The outcome of this study was medical disputes, which were considered as disputes arising during medical practices between patients or their immediate relatives and physicians or medical institutions because of differing perceptions of treatment outcomes. To identify the characteristics associated with medical disputes, the significant features identified through multivariate analysis were used as input features for the ML model. We used 6 techniques—LR, DT, RF, SVM, GBDT, and DNN—in this study [22-26], which are the most commonly used techniques for binary classification predictions. These ML techniques were carefully selected to ensure a comprehensive evaluation. More details regarding these techniques can be found in Multimedia Appendix 1. Learning curves were presented before and after model training to test the overfitting and underfitting of the models. The optimal hyperparameters for each model were determined using a random hyperparameter search.

Validation of the models was conducted using both internal and external sets. We used 12 metrics to evaluate the prediction performance of the models: mean predicted probability, Brier score, intercept, calibration slope, area under the curve (AUC), discrimination slope, specificity, sensitivity, negative predictive value, precision (positive predictive value), Youden index, and accuracy. The equations used to calculate the Brier score, specificity, sensitivity, negative predictive value, precision, Youden index, and accuracy are presented in Multimedia Appendix 1. A scoring system was developed to select the optimal model, where higher scores indicated better predictive performance. In this scoring system, the best performance in each metric was assigned 6 points, scores were given in descending order, and the model with the highest sum of scores across all metrics was considered the optimal model. Furthermore, the clinical usefulness of the models was assessed using a decision curve analysis.

### Model Explainability

After obtaining the optimal model through the predictive evaluation, model explainability was assessed using a local interpretable model-agnostic explanation (LIME) to enhance clinical utility and transparency [24,27]. LIME was able to present the risk probability of encountering medical disputes in individual cases and provide insights into the factors contributing to the predicted risk. By assigning individual weights to each feature, individualized predictions of medical disputes were achieved by calculating the weighted sum of the different features. In addition, Shapley additive explanations (SHAP) was used to analyze the importance of each feature [28]. Further information on SHAP can be found in Multimedia Appendix 1.

### Development of Web-Based Calculator

To enhance the utility and explainability of the model, a web-based app was developed based on *Streamlit*. Detailed information on the development of the web-based calculator is provided in Multimedia Appendix 1. The web-based app allows users to assess the risk of experiencing medical disputes for specific cases by selecting model parameters and clicking the *Submit* button. The calculator includes an introduction to the model, guidelines for users, and risk stratification criteria for users.

### Statistical Analysis

A comparison of features between medical workers with and without medical disputes was conducted using the chi-square test and continuous adjusted chi-square test. Multiple LR was used to identify the significant features associated with medical disputes. Reclassification of patients was performed based on the threshold in the optimal model, and additional information is presented in Multimedia Appendix 1. All ML and model explainability procedures were implemented using Python software (version 3.9.7; Python Software Foundation). Data visualization and statistical analyses were performed using the R programming language (version 4.1.2; R Foundation for Statistical Computing). Statistical significance was determined by a *P* value of <.05 (2 tailed).

### Ethics Approval

The study was approved by both the Hunan Provincial Health Commission (number 2021-17) and the Beijing Health Commission.

### Informed Consent and Participation

All participating hospitals fully understood and supported the entire content of the survey. Participation in the survey was voluntary, and all participants provided informed consent before participating. The survey was anonymous and did not collect any personal information from the participants. This study adhered to the principles outlined in the Declaration of Helsinki.

## Results

### Baseline Characteristics and Status of Hospital Legal Construction

Most of the enrolled medical workers worked in public hospitals (36,122/38,053, 94.93%), general hospitals (24,189/38,053, 63.57%), and class A tertiary hospitals (21,642/38,053, 56.87%; Table S1 in Multimedia Appendix 2). Physicians and nurses accounted for 39.36% (14,977/38,053) and 41.63% (15,843/38,053) of the participants, respectively. In terms of technical titles, 40.23% (15,308/38,053) of the participants were middle technical title holders, followed by junior title holders (13,440/38,053, 35.32%). Among all enrolled medical workers, 44.79% (17,044/38,053) were in the age range of 30 to 39 years, and 71.03% (27,029/38,053) were female. Regarding hospital legal construction, 95.77% (36,443/38,053) of the medical workers reported that their hospitals had already established hospital legal construction. In addition, 71.2% (27,093/38,053) had an independent rule of law department, and 76.71% (29,190/38,053) mentioned that their hospital performance appraisal system included items related to hospital legal construction. Among the participants, 93.86% (35,715/38,053) reported that their hospitals organized legal training, 86.59% (32,949/38,053) had examinations on law popularization among hospital staff, and 81.66% (31,074/38,053) provided rule of law training for new recruits. However, 17.85% (6791/38,053) of the participants had never undergone any legal training organized by their hospitals.

Regarding the participants’ knowledge of medical law, most medical workers reported that they had a very clear or clear understanding of the hospital president responsibility system, the duties of the hospital law department, the duties of the legal counselor in the hospital, and the contents of hospital charters. Of the medical workers, 70.48% (26,819/38,053) believed that hospital legal construction was very helpful to their medical work, and 69.27% (26,360/38,053) expressed a strong willingness to participate in the law training organized by the hospital. However, 75.83% (28,857/38,053) of medical workers still considered it very necessary to conduct legal training in their respective hospitals.

These results indicate that although most hospitals had implemented the hospital rule of law construction, the effectiveness of these efforts was not entirely satisfactory because some participants (17.85%, 6791/38,053) had never received any legal training and a substantial number of participants (96.99%, 36,906/38,053). This might partly explain why medical disputes occurred in up to 46.06% (17,527/38,053) of the enrolled medical workers.

### Comparison of Medical Workers Stratified by Medical Disputes

A comparison of characteristics was performed based on the presence or absence of medical disputes among the medical workers. The results revealed differences in the distribution of various clinical characteristics between medical workers with and without medical disputes. These significant clinical characteristics included basic demographics, hospital level, construction of the hospital rule of law, participants’ knowledge of medical laws, and participants’ awareness of medical laws (all *P*<.05). Further details regarding hospital legal construction and participants’ knowledge and awareness of medical laws are summarized in Table S1 in Multimedia Appendix 2. The analysis indicated that being male; having a higher age; holding a senior physician position; working in a public hospital, general hospital, or class A tertiary hospital; experiencing poorer construction of the hospital rule of law; having less knowledge of medical laws; and lack of awareness of medical laws were associated with a higher likelihood of medical disputes.

### ML Modeling

A multivariate analysis identified 18 characteristics that were significantly associated with medical disputes (all *P*>.05; Table S2 in Multimedia Appendix 2). These significant characteristics were used as input features for training the LR model and the 5 ML models. The models were constructed and trained using the training cohort to predict the occurrence of medical disputes among medical workers. Learning curves before and after model training are presented in Multimedia Appendix 1, which demonstrated that overfitting of the models was considerably mitigated after model training. The complete hyperparameters for each model are summarized in Table S3 in Multimedia Appendix 2.

### Internal Validation

**Figure 2:**
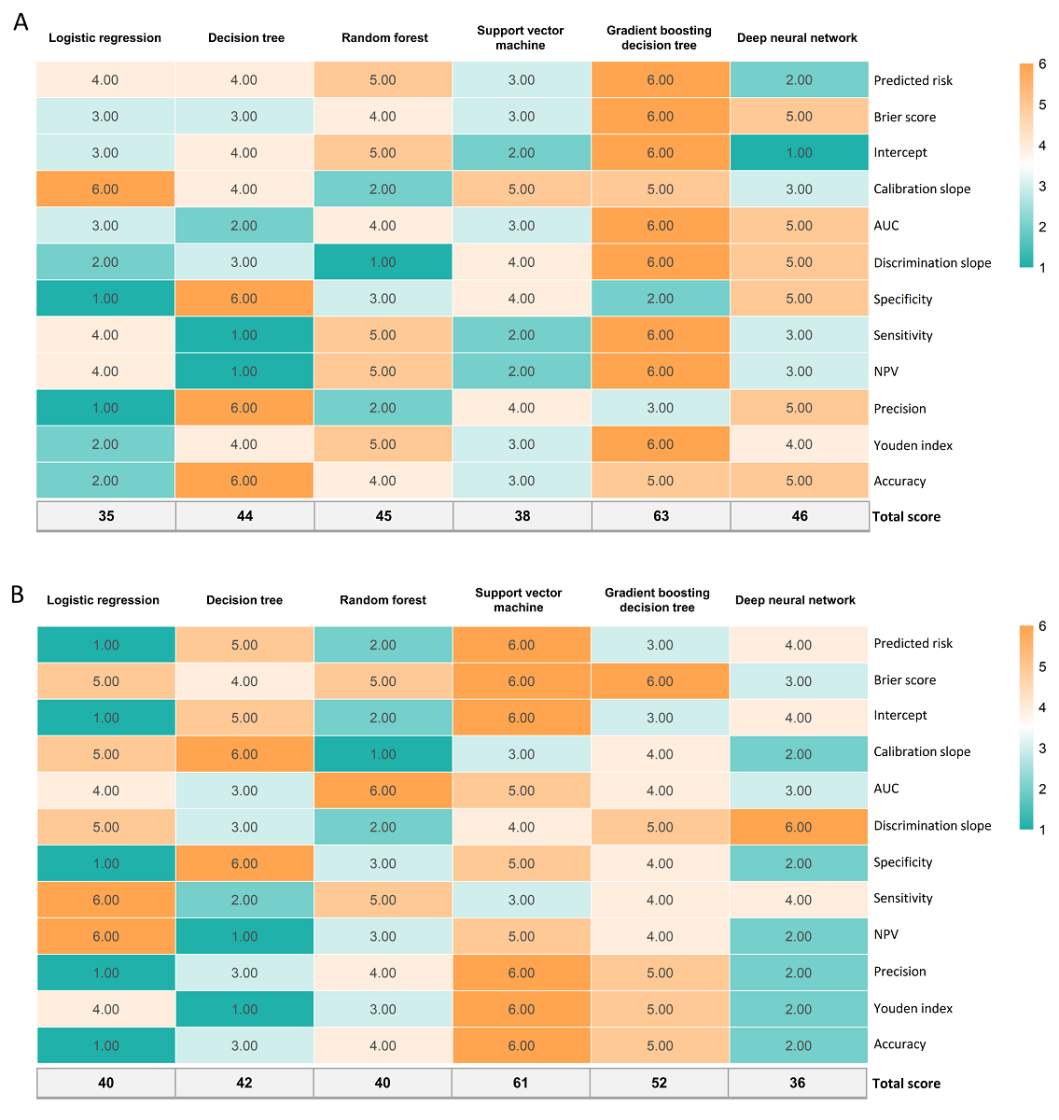
Heat map for the prediction performance of each model in the (A) internal validation set and (B) external validation set after evaluation using a scoring system. Cyan-colored boxes indicate a low value, whereas orange-colored boxes represent a relatively high value. The total score was calculated as the sum of the values from the 12 metrics, and a higher total score indicates better prediction performance. AUC: area under the curve; NPV: negative predictive value.

**Figure 3:**
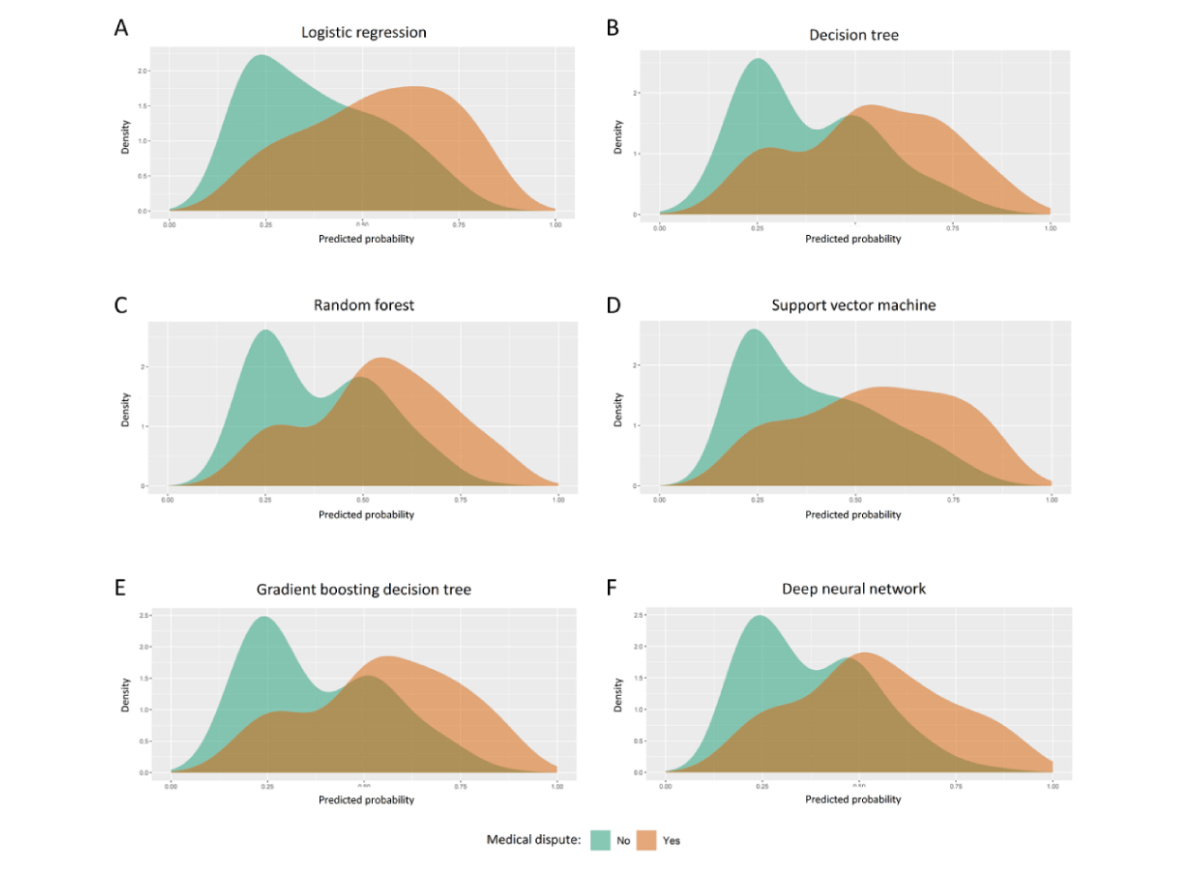
Probability curve for (A) logistic regression, (B) decision tree, (C) random forest, (D) support vector machine, (E) gradient boosting decision tree, and (F) deep neural network in the internal validation set. The probability curve was drawn with predicted probability against density. Cyan color indicates participants without medical disputes, and orange color indicates participants with medical disputes.

On the basis of the scoring system used to evaluate the metrics in the study, among all 6 models, GBDT exhibited the best prediction performance with the highest score (63 points), followed by DNN (46 points) and RF (45 points) in the internal validation set (Figure 2A). Specifically, GBDT achieved the highest AUC value (AUC=0.738; Figure S1 in Multimedia Appendix 3; Table 1), followed closely by DNN (AUC=0.734). Probability curves were plotted for each algorithm (Figure 3). The ML models, particularly GBDT and DNN, demonstrated large separation of the curves with minimal overlap and substantial distinction between the 2 groups compared with the other techniques. To provide quantitative analysis, violin plots were generated (Figure S2 in Multimedia Appendix 3), and discrimination slopes were calculated. GBDT exhibited the highest discrimination slope (0.172), followed by DNN (0.164) and SVM (0.160). The calibration curves for each algorithm are shown in Figure S3 in Multimedia Appendix 3, indicating favorable consistency between the predicted and observed probability of medical disputes across most models. Notably, GBDT demonstrated excellent calibration, with a calibration slope very close to 1 and an intercept very close to 0. The decision curve analyses for each algorithm are presented in Figure S4 in Multimedia Appendix 3.

**Table 1:** Prediction performance of machine learning approaches for predicting medical disputes among medical workers in the internal validation group.

Measures	Approaches					
	Logistic regression	Decision tree	Random forest	Support vector machine	Gradient boosting decision tree	Deep neural network
Actual risk (%)	45.87	45.87	45.87	45.87	45.87	45.87
Predicted risk (%)	45.69	45.69	45.77	46.52	45.79	44.94
Brier score	0.210	0.210	0.207	0.210	0.205	0.206
Intercept	0.009	0.008	0.005	−0.031	0.004	0.045
Calibration slope	1.001	0.971	1.127	0.976	1.024	1.056
Area under the curve (95% CI)	0.726 (0.710-0.742)	0.725 (0.708-0.741)	0.733 (0.717-0.749)	0.726 (0.710-0.742)	0.738 (0.722-0.754)	0.734 (0.718-0.749)
Discrimination slope	0.156	0.158	0.152	0.160	0.172	0.164
Specificity	0.651	0.722	0.659	0.701	0.653	0.705
Sensitivity	0.682	0.622	0.689	0.638	0.701	0.639
Negative predictive value	0.707	0.693	0.714	0.695	0.721	0.697
Precision (positive predictive value)	0.623	0.655	0.631	0.644	0.632	0.647
Youden index	1.333	1.344	1.348	1.339	1.355	1.344
Accuracy	0.665	0.676	0.673	0.672	0.675	0.675
Threshold	0.448	0.500	0.472	0.483	0.466	0.463

### External Validation

In the external validation set, medical disputes were observed in 34.11% (8967/26,285) of the medical workers. Table S4 in Multimedia Appendix 2 presents the baseline characteristics of the patients and a comparison between medical workers with and without medical disputes. We observed a significant difference in the 18 model features stratified by medical disputes, which was consistent with the findings in the Hunan database. This indicates that the 18 model features were reliable predictors, as their significance in medical disputes was confirmed in the external validation. However, the predictor, participating in legal training organized by hospitals, was only marginally significant (*P*=.07). In the external validation cohort, the prevalence of medical disputes was 34.1%, compared with 46.06% in the Hunan database. The difference in the prevalence of medical disputes between the internal and external sets can be attributed to the heterogeneity of the geographical regions and populations. In addition, the proportion of medical workers who had previously faced legal issues outside of medical work was only 11.84% (3111/26,285) in the external validation set, whereas this figure was as high as 52.51% (19,983/38,053) in the Hunan database.

A favorable prediction performance of the models was also achieved in the external validation set based on the 12 metrics (Table S5 in Multimedia Appendix 2). Specifically, among the 6 techniques, SVM showed the best prediction performance with the highest metric score (61 points), followed by GBDT (52 points) and DT (42 points) in the external validation set (Figure 2B). The ranking of the prediction performance of SVM improved from fifth in the internal validation set to first in the external validation set, indicating its instability in our study. Thus, SVM was not considered as the optimal model, despite its excellent prediction performance in the external validation set. In contrast, GBDT outperformed the other models in the internal validation set and maintained its ranking as second in the external validation set. Therefore, the GBDT model was deemed the optimal model in this study. In the external validation set, the model’s performance in terms of area under the receiver operating curves, violin plots for discrimination slopes, calibration curves, and decision curves is summarized in Multimedia Appendix 1.

### Model Explainability

**Figure 4:**
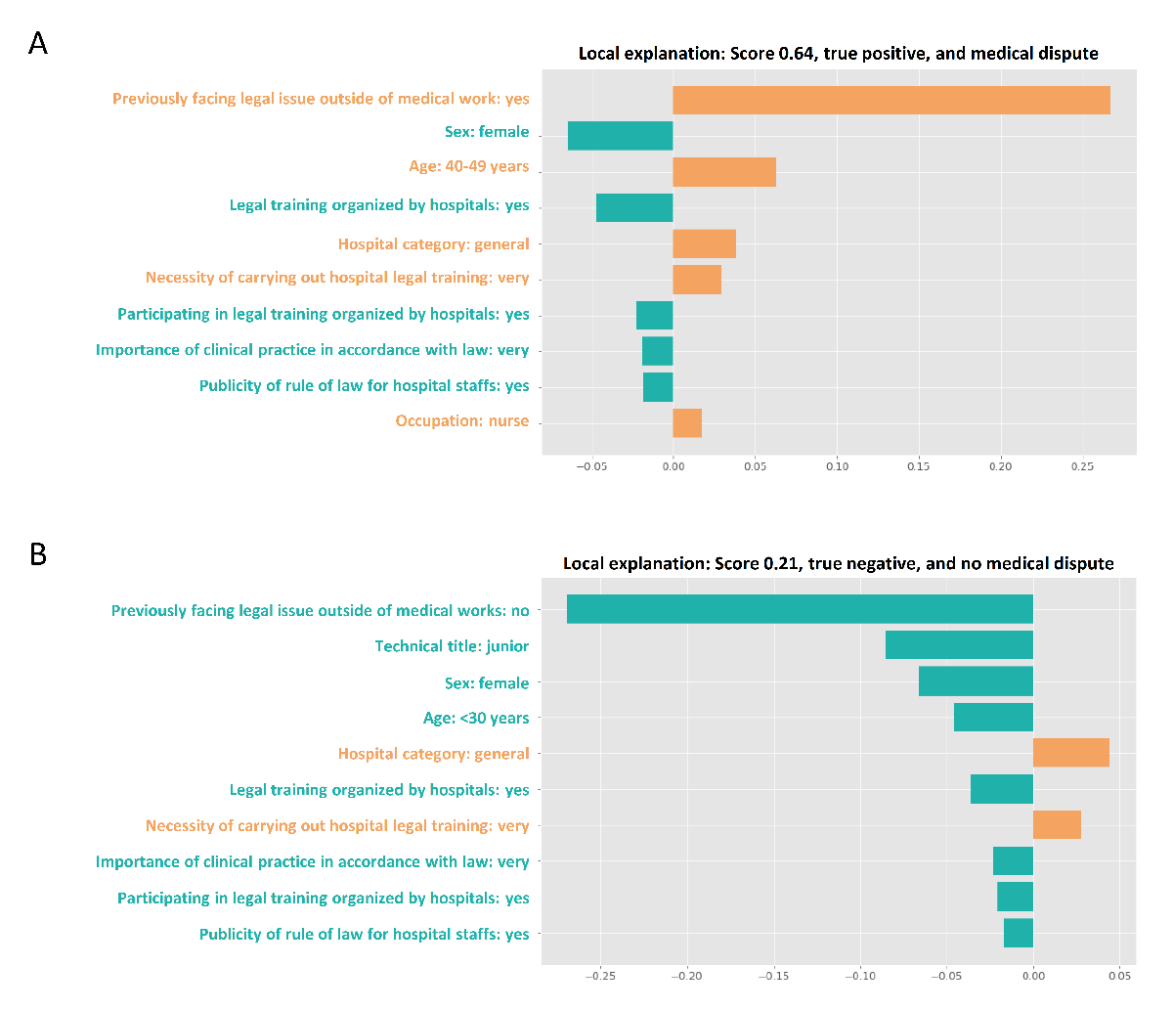
Model explainability using local interpretable model-agnostic explanation: (A) a true positive case and (B) a true negative case. In the first case, the study depicted a specific individual with a high probability of experiencing medical dispute (64%), whereas the second case showed low risk of medical dispute (21%). Features with an orange bar imply contributory elements to increase medical dispute, whereas those with a cyan bar indicate protective features.

In this study, LIME was used to assess the model explainability for the optimal model generated by GBDT. LIME was able to rank feature importance and visualize their contributions to medical disputes on an individual case basis. The top 10 features for each case were identified (Figure 4), with the weight of each feature presented in either cyan (indicating the prevention of medical disputes) or orange (representing the promotion of medical disputes) in the figure. The study presented a true positive (Figure 4A) and true negative (Figure 4B) case.

In this study, a heat map was used to visualize the SHAP values of the first 1000 participants in the internal (Figure S5A in Multimedia Appendix 3) and external (Figure S5B in Multimedia Appendix 3) validation sets. The heat map revealed the top 10 important features for the 2 sets, which identified previously facing legal issues outside of medical work, technical title, occupation, sex, and age as the top 5 most important features in both the sets. Feature importance was also assessed for the entire internal and external validation sets, which demonstrated similar results (Figures S6A and S7A in Multimedia Appendix 3). In addition, the mean SHAP values for the top 10 important features were summarized in the entire internal (Figure S6B in Multimedia Appendix 3) and external (Figure S7B in Multimedia Appendix 3) validation sets.

### Web-Based Calculator

We established a web-based calculator, which is freely accessible on the internet [29]. On accessing the link, researchers can use the web-based calculator after selecting relevant features from the panel of parameters. Next, the probability of medical disputes can be automatically calculated by clicking the *Submit* button. In addition, corresponding suggestions on preventing medical disputes are provided in terms of risk classification to implement individualized preventive strategies. Participants with a predicted probability at or below the threshold value (≤46.6%) were classified as the low-risk group, whereas those with a predicted probability above the threshold (>46.6%) were classified as the high-risk group (Table S6 in Multimedia Appendix 2). Participants in the high-risk group had up to twice the odds of experiencing medical disputes compared with those in the low-risk group (*P*<.001). A screenshot of the web-based calculator is shown in Figure S8 in Multimedia Appendix 3. Notably, at times, if the web-based calculator becomes inactive and cannot be accessed (the following screenshot), users can revive it by clicking on the *Yes, get this app back up!* button. Within approximately 30 seconds, the web-based calculator will be accessible once again after booting the app.

When it comes to model explanation in ML, there are several techniques available such as LIME, SHAP, and web-based calculator, as mentioned earlier. Each of these methods has advantages and disadvantages. However, after careful consideration, the web-based calculator was chosen as the preferred option in this study; more detailed information is summarized in Multimedia Appendix 1.

## Discussion

### Principal Findings

The developed prediction model can stratify medical workers into different risk groups when encountering medical disputes. This provides a valuable tool for identifying high-risk individuals and implementing preventive strategies. In addition, the model was externally validated using data from medical workers in Beijing, further enhancing its reliability and generalizability. Notably, this study’s impact extends beyond research, as it created a web-based app for medical dispute prediction. This user-friendly tool can be accessed by the public, thereby facilitating its practical app in clinical settings. By inputting relevant characteristics, health care workers and stakeholders can evaluate the risk of medical disputes for individual cases.

A nationwide survey conducted in China in 2021 revealed that 31.06% of physicians had dealt with medical disputes involving patients [30], and a meta-analysis conducted in the same year revealed that 55.73% of medical professionals thought that the physician-patient relationship was stressful [31]. Both findings demonstrated that medical disputes were prevalent. In this study, medical disputes occurred in 46.06% (17,527/38,053) of medical workers in Hunan province and 34.11% (8967/26,285) in Beijing, which was consistent with the findings of other studies [3,4]. According to the current literature, a nationwide cross-sectional survey conducted in 2020 showed that 33.48% of physicians experienced medical disputes among medical workers [3], and 76% of medical workers encountered workplace violence [4]. The difference in the prevalence of medical disputes among the different data sets could be explained by the heterogeneity of the geographical regions and populations.

Preventive strategies are urgently needed to address medical disputes. To implement personalized preventive strategies, we developed a novel prediction model to classify medical workers into different risk categories based on their likelihood of experiencing medical disputes in the upcoming year. We proposed several models using ML techniques and introduced a wide selection of ML techniques for the analysis. GBDT demonstrated superior performance, achieving the highest score in the internal validation set and ranking second in the external validation set. A single indicator alone has limited predictive value for medical disputes. However, by combining multiple indicators, the predictive performance is greatly improved. In this study, we identified 18 features that were significantly associated with medical disputes and used them as the input features for the model. Through ML modeling, favorable prediction effectiveness was achieved, as demonstrated by calibration and discrimination metrics. ML prediction models offer several advantages over traditional models because they can effectively handle large and complex data sets, identify nonlinear relationships, and improve prediction accuracy. Furthermore, ML models can be trained on a subset of data and validated on an independent subset, thereby reducing the risk of overfitting. Consequently, the GBDT model outperformed the traditional LR model in terms of prediction performance in this study.

Although previous studies have established prediction models such as using medical claims data to predict suicide risk [32], forecasting future health care use and combining longitudinal claims data and clinical context information to forecast drug-related hazards [33], and estimating the likelihood that patients would fall into the top 10% of cost distribution in the upcoming year [34], our prediction model is the first to predict medical disputes specifically among medical workers in a very large sample. Previously, our team developed a model to evaluate the risk of medical disputes, especially among hospital administrators, using traditional LR analysis after analyzing 2716 administrators [12]. However, that study did not use ML techniques, and the AUC of the model was 0.68.

To determine the best-performing model among the 6 models, we compared their predictive performance using a scoring system to rank the techniques in terms of predictive effectiveness. The prediction performance mainly assessed discrimination and calibration, both of which are crucial aspects of prediction performance [35,36]. Previous studies have reported that the performance of models could not be sufficiently revealed using commonly reported metrics [35], highlighting the need for a comprehensive evaluation of performance metrics when reporting prediction models. The scoring system used in this study allowed for a comprehensive assessment of the prediction performance of the models by incorporating 12 metrics. Among the 6 models, the GBDT model emerged as the optimal model. Therefore, this study used the SHAP and LIME explainers to analyze the model’s output and determine feature importance. Notably, LIME had the advantages of displaying specific risk probabilities of medical disputes and elucidating the underlying mechanisms [24]. Displaying the model on the internet would further promote the utility of the model because it would be considerably convenient and simple for medical workers to use; thus, we finally developed a web-based app to present the optimal model.

This study demonstrated a significant association between the 18 characteristics and medical disputes. Specifically, working in a public hospital, general hospital, or class A tertiary hospital; being a physician; having a senior technical title; being male and of older age; lacking concern for the duty of the hospital law department or legal counselor; not having a clear understanding of the contents of hospital charters; considering it less important to conduct clinical practice in accordance with the law; having a strong need for legal training in hospitals; and having previous legal issues outside of medical work were identified as predictors of medical disputes. Conversely, legal training organized by hospitals, having publicity of the rule of law for hospital staff, participating in legal training organized by hospitals, being willing to participate in law training organized by hospitals, and having a well-established rule of law in the hospital were found to be protective factors. These findings are consistent with the current literature, which also elucidated that conducting standardized high-level hospitals [37], providing preservice skills training [38], having older physicians [10], male physicians [10,11,39], a higher professional level [11], and implementing specific and appropriate laws and regulations [40] were significantly associated with medical disputes. Higher-ranked physicians often face heavier workload, which can adversely affect their health and the quality of their services [11]. Patients have higher expectations of physicians with higher ranks and tend to seek high-level medical services even for minor and self-limiting illnesses. Consequently, the increased patient load increases the work pressure on physicians, leading to long-term overwork. This, in turn, can contribute to rushed interactions, indifference, and disrespect toward patients, which are major causes of physician-patient disputes [41]. Chinese medical workers, particularly those in top-tier public institutions, often experience excessive workload, occupational stress, and burnout syndrome [37]. This may explain why public, general, and class A tertiary hospitals have a higher incidence of medical disputes. In addition, medical workers in these hospitals were more likely to treat patients with more serious illnesses, which may be another contributing factor [37].

This study primarily focused on investigating the relationship between medical disputes and hospital legal construction, providing valuable supplementary findings to the current literature. We demonstrated that possessing good knowledge about medical law was conducive to reducing medical disputes among medical staff. Publicizing the rule of law for hospital staff and encouraging their participation in legal training courses were identified as greatly beneficial in preventing medical disputes. In addition, up to 52.51% (19,983/38,053) of the medical workers in the Hunan data set reported having previously experienced legal issues outside of medical work in the Hunan data set. This finding suggests that hospitals should prioritize cultivating legal literacy among their staff and develop strategies to prevent and address legal problems even if these legal problems were not related to medical work.

Other studies have already proposed helpful suggestions for the prevention of medical disputes. For instance, an integrative review conducted by Jack et al [42] pointed out that a physician’s ability to build trust with patients, increase patient satisfaction, and provide high-quality care was aided by effective communication and interpersonal skills. Yu et al [43] highlighted the advantages of establishing efficient quality control mechanisms for medical records to reduce medical disputes. Liu et al [44] found that lower medical costs were helpful in reducing conflicts. Lan et al [45] found that increasing the private sector in the health care market could alleviate medical disputes through the competition mechanism. In this study, we further emphasized the importance of publicizing the rule of law for hospital staff. Reducing medical disputes and enhancing physician-patient relationships require systematic changes over a sustained period [46]. First, it was important to organize legal training for hospital staff, invite legal specialists to give lectures at the hospital on legal education and case studies for all staff members, provide uniform pre-employment training for all majors, practice according to management measures and technical guidelines, and practice adhering to the law [38]. Second, hospital workers should be incentivized to participate in legal education programs. Hospitals should develop compelling strategies to entice physicians to engage in legal training in hospitals, such as providing clear instructions for managing training, issuing certificates for continuing education, and offering web-based instructions regarding hospital rules and regulations. Finally, the government should formulate laws and regulations, strengthen cooperation between hospitals and public security departments and people’s mediation organizations, and establish effective working mechanisms. By revising the laws and regulations, improving the mechanism of third-party mediation of medical disputes, and implementing third-party compensation for medical liability insurance, a firewall for handling medical disputes between physicians and patients can be established. Furthermore, health administrative departments and medical institutions should demonstrate a positive mental state and a strong sense of responsibility to implement the relevant laws and regulations. In this study, based on risk stratification, participants in the high-risk group were found to have twice the odds of experiencing medical disputes compared with those in the low-risk group. Therefore, the aforementioned preventive strategies should be particularly emphasized in the high-risk group. This means that medical workers in the high-risk group require more extensive legal training and comprehensive knowledge of medical laws. Importantly, in China, there is a serious shortage of investment in medical health care, with only 3% of the world’s total health expenditure being spent on approximately 20% of the world’s population [47]. Therefore, policies aimed at increasing health spending and optimizing the allocation of medical resources are warranted to prevent and address medical disputes, especially in low-income countries and countries with limited health care investment. In addition, promoting a clear understanding of medical knowledge among patients can also help in resolving disagreements [48], highlighting the importance of popularizing medical knowledge.

### Limitations

This study had some limitations. First, when completing the questionnaire, physicians were susceptible to have recall bias, which may lead to inaccuracies in the information provided. However, the reliability of the questionnaire was confirmed in this study. Second, several studies have reported that some other characteristics such as personality traits and mental health status were relevant factors in medical disputes [49]. Nonetheless, this study collected approximately 30 characteristics and focused primarily on investigating the relationship between hospital legal construction and medical disputes. Third, the prediction model was not internationally validated, limiting its generalizability to international data sets and warranting further investigation. Therefore, although the model was developed based on ML and validated using a large multicenter data set, extensive validation in international data sets is warranted.

### Conclusions

Medical disputes are a prevalent issue among medical workers. Among the models compared, GBDT demonstrated a promising predictive performance. This web-based tool serves as a screening tool to identify medical workers who are at a higher risk of experiencing medical disputes. Preventive strategies should be emphasized, particularly in the high-risk group. Enhancing the knowledge and awareness of the medical law among medical workers can be effective in preventing medical disputes.

## Appendix

Multimedia Appendix 1 Methods, results, and references of the study.

Multimedia Appendix 2 A comprehensive analysis, comparing the characteristics of participants with and without medical disputes in both the Hunan and Beijing databases, the multivariate analysis to identify the key characteristics that predict medical disputes in the training group, the machine learning-based approaches and hyperparameters, the prediction performance of these machine learning approaches in the external validation group, and the risk stratification, validated internally and externally, to effectively identify individuals at different risk levels for medical disputes.

Multimedia Appendix 3 The area under the receiver operating curve, violin plots for the discrimination slope, calibration curves, decision curve analysis, the heatmap of Shapley additive explanations (SHAP) values in the internal validation set, analysis of feature importance based on SHAP summary plot in the internal and external validation set, and the web-based application.

## Abbreviations

AUC: area under the curve
DNN: deep neural network
DT: decision tree
GBDT: gradient boosting decision tree
LIME: local interpretable model-agnostic explanation
LR: logistic regression
ML: machine learning
RF: random forest
SHAP: Shapley additive explanations
SVM: support vector machine

## Data Availability

On reasonable request and with the approval of the Hunan Provincial Health Commission and Beijing Municipal Health Commission, data may be obtained from the corresponding author.
