# Supplementary material for: Prediction of Medical Disputes Between Health Care Workers and Patients in Terms of Hospital Legal Construction Using Machine Learning Techniques: Externally Validated Cross-Sectional Study
Source: J Med Internet Res. 2023 Aug 17;25:e46854. doi: 10.2196/46854 (PMC10472173; doi:10.2196/46854)
Supplement: Multimedia Appendix 1 [file jmir_v25i1e46854_app1.docx]

**Supplementary file**

**Characteristics and Prediction of Medical Disputes Between Health Care Workers and Patients in Terms of Hospital Legal Construction Using Machine Learning Techniques: Findings From an Externally Validated Cross-Sectional Study**

**Table of Contents**1

**Methods2-12**

Sampling methods2

Definition of hospital legal construction and other clinical characteristics2-5

A brief introduction of machine learning techniques5-9

Supplementary contents of performance assessment10-12

Supplementary contents of feature importance12

Development of online calculator12

Risk stratification system according to the optimal model12

**Results13-21**

Learning curves13-17

Area under the receiver operating curve in the external validation set 18

Violin plots for the discrimination slopes in the external validation set 19

Calibration curves in the external validation set 20

Decision curve analysis in the external validation set 21

A comparison of model explanation using LIME, SHAP, and online calculator 22-24

**Supplementary references25-26**

**Methods**

**Sampling methods**

The questionnaire for medical staff in Hunan Province was distributed to 130 tertiary hospitals in Hunan Province, relying on the Health Commission of Hunan Province. Each hospital should roughly distribute about 300 questionnaires to medical staff at random, with a 4:4:1:1 ratio of doctors, nurses, pharmacists, and technicians in terms of the Health Statistics Yearbook in China. Priority is given to ensuring the number of questionnaires collected, and the proportion of survey respondents is met on the basis of meeting the number. The questionnaires for medical personnel in Beijing were distributed to 87 hospitals in Beijing by the Beijing Municipal Health Commission, and about 500 medical staff were randomly selected from each institution, with the similar proportion of doctors, nurses, pharmacists, and technicians with Hunan Province.

Administrative staff of hospitals received the survey from the local health commission and then assigned the survey to medical workers. Medical workers were voluntary to participant in the survey, and the survey could be submitted only when all questions were completed based on their own actual status. Under such circumstance, the integrity and the quality of collected data could be considerably ensured.

**Definition of** **hospital legal construction and other clinical characteristics**

**Hospital legal construction**

Hospital legal construction was proposed by the National Health Commission of China in 2019 (1), and it aims to strengthen the rule of law construction of medical and health institutions with the contents of enhancing legal thinking and capabilities among medical workers, improving the system of rule of law construction, gradually establishing and improving the rule of law working department, and implementing the legal adviser system.

**Other clinical characteristics**

| The definition of clinical characteristic. | |
| --- | --- |
| Characteristics | Definition |
|  |  |
| Hospital type (Public/Private, %) | A public hospital is a hospital which is owned by a government and receives government funding.  A private hospital is a hospital owned by a profit company or a non-profit organization and privately funded through payment for medical services by patients themselves, by insurers, or by Governments through national health insurance schemes. |
| Hospital category (%) |  |
| General | General hospital is a hospital designed to deal with a wide range of illnesses and injuries, and it often includes emergency department, outpatient clinic and inpatient unit. General hospitals are usually the primary medical facility in an area, with a large number of beds that can provide intensive care and long-term care for many patients at once. |
| Traditional Chinese Medicine | Traditional Chinese Medicine (TCM) is a comprehensive medical health care system comprised of a range of traditional therapies including, but not limited to: Acupuncture, acupressure, moxibustion, herbal medicine, nutrition, tui na massage and exercises (tai chi and qigong). |
| Integrated Chinese and western medicine | Integrated Chinese and Western medicine is the intersection of Chinese and Western medicine and a working policy in Chinese medical and health care. It is the combination of traditional Chinese medicine knowledge and methods with Western medicine knowledge and methods to improve clinical efficacy, elucidate the mechanism and thus gain new medical understanding. |
| National | The National Hospital has gathered the essence of medicine from all major ethnic minorities in China, including Tibetan medicine from the snowy plateau, Mongolian medicine from the steppes of Inner Mongolia, Yi medicine from the frontier of Yunnan, Tujia medicine from Xiangxi, Hunan, Zhuang medicine from Guangxi Zhuang Autonomous Region, Buyi medicine from Guizhou, and ethnic medicine specialties such as Chinese medicine. |
| Specialized | A Specialized Hospital is a hospital that specializes in only one or a few subspecialties of medicine. |
| Maternity and child healthcare hospital | Maternity and child healthcare hospital refers to specialized medical institution that provides medical services exclusively for women and children. |
| Others |  |
| Tertiary hospital level (%) | Tertiary hospitals are hospitals that provide medical and health services across regions, provinces and cities as well as to the whole country, and are medical and preventive technology centers with comprehensive medical, teaching and research capabilities. |
| Occupation | Occupation refers to the occupation in which the respondent was employed at the time the questionnaire was completed. |
| Technical title | The Technical title is the professional and technical rank that the respondent had at the time of filling out the questionnaire. |
| Establishment of hospital legal construction | The construction of the rule of law is a national system for the common management of state affairs within a certain class range and in accordance with the principles of equality and minority obedience to majority within the framework of the Constitution and laws. The specific performance is to improve legislation, strict law enforcement, and vigorously popularize the law. The hospital implements the basic governance strategy of "rule by law" and manages hospital affairs in accordance with the principles of rule of law construction. |
| Understanding hospital president responsibility system under the leadership of the hospital Party committee | Party Committee is the abbreviation of the Committee of the Communist Party of China at all levels. The President's system of responsibility under the leadership of the Party Committee refers to the implementation of a combination of collective leadership and individual responsibility for the division of labor, all major issues in accordance with the principle of collective leadership, democratic centralization, individual brewing, meeting decisions, the Party Committee to discuss collectively, make decisions, and in accordance with the division of labor to grasp the organization and implementation, and support the President to exercise his powers independently and responsibly in accordance with the law. |
| Independent rule of law department in the hospital | The hospital set up a rule of law construction department, matched with appropriate staff and funding to carry out the corresponding rule of law construction work. |
| Understanding the duty of hospital law department | The main responsibilities of the hospital rule of law department are: to participate in decision-making on major hospital matters; to participate in hospital system construction; to sort out hospital legal risk points; to supervise and inspect the implementation of the rule of law construction tasks; to cooperate with supervision and inspection; " Popularization of law" and legal policy knowledge training and education; provide legal services for the hospital departments and staff; contact management of legal counsel and undertake other matters assigned by the hospital. |
| Understanding the duty of legal counselor in the hospital | The main responsibilities of the hospital's legal counsel are: to review the legality of important hospital documents, contracts and law-related documents; to participate in handling law-related disputes and negotiations; and to represent the hospital in litigation and arbitration. |
| Understanding the contents of hospital charters | Hospital charters are hospital law frameworks that include state law, administrative regulations, and hospital regulations and rules. |
| Hospital performance appraisal system including hospital legal construction | Performance appraisal system including hospital legal construction means that hospital legal construction was regarded as an important item of performance appraisal system. |
| Importance of clinical practice in accordance with law | Clinical practice according to the law requires doctors to strictly abide by legal norms and follow medical ethics in the process of treating patients and saving lives. Operating within the limits of the law, they should not lose their basic quality of medical practice and ethics due to private interests. |
| Necessity of carrying out hospital legal training | The rule of law training carried out by the hospital mainly includes: rule of law training for new recruits; daily rule of law study for employees, such as lectures and training; and general law examinations for employees. |
| Previously facing legal issue outside of medical works (No/Yes, %) | Encounter legal disputes in daily life. |

**A brief introduction of machine learning techniques**

**Logistic regression (LR)**

LR utilizes a logistic function to distill a linear equation’s output between negative (‘0’) and positive (‘1’) outcomes. Feature weights are unable to be explained as linear regressions because they do not linearly impact the probability. A good approach to assess feature weights in terms of logistic regression depends on treating it as a linear model for the log odds. Users is able to calculate the odds ratio for each feature by obtaining the corresponding weight exponent (2). Logistic function is depicted below:

$$\sigma(x)=\frac{1}{1+e^{-x}}$$

Among them, the parameters of LR model includes weight $\vec{w}$ and intercept $b$, and thus it can be also presented as $\vec{\theta}=(\vec{w}\cdot\vec{x}+b)$. $\vec{x}$ is the input variable, and LR model is presented as the following equation:

$$f\left( \vec{x};\vec{\theta} \right)=\sigma(\vec{w}\cdot\vec{x}+b)$$

The entire number of parameters of the logistic model is $d+1$, and $d$ indicates the weight of $\vec{w}$ and 1 indicates intercept. The parameters of the logistic regression $\vec{\theta}$ can be fitted using the maximum likelihood estimation. The output probability of the logistic model is:

$$p_{i}=f\left( \vec{x_{i}};\vec{\theta} \right)$$

**Decision tree (DT)**

DT is a tree-structured scheme, in which nodes represent the input features and leaves symbolize decision outcomes (3). It is one of the earliest and most prominent ML techniques, and it has proven to be a valuable tool for extracting meaningful information from measured data and represent a plausible solution for massive data learning tasks (4).

If there are $C$ categories of data in sample dataset $S$. The Gini index is presented as the following equation (5):

$$Gini\left( S \right)=1=\sum_{i=1}^{c} P_{i}^{2}$$

Where in the study $S$ indicates the training set, $C$ indicates the data class number, and $P_{i}$ indicates the ratio of the sample number in class $i$ to all samples. If the current node corresponds to the training set $S$, and characteristic root $v$ classifies $S$ into $k$ disjoint subsets $S_{1}$, $S_{2}$, $S_{3}$, …, $S_{k}$, that is:

$$S=S_{1}\cup S_{2}\cup S_{3}\cup\ldots S_{k}$$

Thus, the information gain $G\left( S, v \right)$ is presented as follows:

$$G\left( S, v \right)=Gini\left( S \right)-\sum_{i=1}^{k} \frac{\left| S_{i} \right|}{\left| S \right|}Gini(S_{i})$$

**Random forest (RF)**

RF is a combined classifier consisting of many tree-structured classifiers (6), hence the name is ‘forest’. It is an ensemble learning algorithm, which are aggregated through bootstrap (bagging), and it predicts a sample by combining the predictions from all individual decision trees via averaging or a majority vote (7). Compared to a single decision tree model, a random forest model exhibits less overfitting, especially when working with a small dataset.

If the $T$ trees of RF are indicated by $\theta_{1}$, $\theta_{2}$,…, $\theta_{T}$, and $w_{i}(x)$ is the average weight. The average RF prediction for the sample $x$ can be presented by weighted average of predictions of all $T$ trees utilizing the weight vector:

$$w_{i}\left( x \right)=\frac{1}{T}\sum_{j=1}^{T} w_{i}(x,\theta_{j})$$

$$\hat{y}\left( x \right)=\sum_{i=1}^{n} w_{i}(x)y(i)$$

**Support vector machine (SVM)**

SVM is a more recent model of ML techniques. It a supervised learning algorithm, defining a discrimination classifier via a separating hyperplane which is capable of maximizing the margin between negative and positive events (3). SVMs first translate the input vector into a higher-dimensional feature space and locate the hyperplane that divides the data points into two classes. The instances that are closest to the border have the greatest marginal distance from the decision hyperplane. The generated classifier has a high degree of generalizability and may be used to accurately classify fresh samples. The formula presenting the squared correlation coefficient ($r^{2}$) of SVM is written as (8):

$$r^{2}=\frac{(\bar{I}\sum_{i=1}^{\bar{I}} f(x_{i})y_{i}-(\sum_{i=1}^{\bar{I}} {f(x_{i})\sum_{i=1}^{\bar{I}} y_{i})}^{2}}{(\bar{I}\sum_{i=1}^{\bar{I}} {f(x_{i})}^{2}-(\sum_{i=1}^{\bar{I}} {f(x_{i}))}^{2})(\bar{I}\sum_{i=1}^{\bar{I}} {y_{i}}^{2}-(\sum_{i=1}^{\bar{I}} {y_{i})}^{2})}$$

**Gradient boosting decision tree (GBDT)**

GBDT is an ensemble learning method, and it is able to integrate weak learners in a strong one so that the loss function decreases gradually, establishing additional base-learners in an iterative manner, which have a maximal correlation with the negative slope of a cost function (7). It is widely used due to its effectiveness, and its prediction performance is superior to the logistic regression model (9).

The GBDT technique utilizes the decision tree as weak learners:

$$T\left( \vec{x};\vec{\theta} \right)=\sum_{j=1}^{J} \gamma_{j}I(\vec{x}{\in R}_{j})$$

Among them, $j$ represents the number of leaves, and the disjoint sections $R_{j}$ determine it$.$ Each region’s values are represented by $\gamma_{j}$. $\vec{\theta}$ represents a collection of parameters of the decision tree, $I(\vec{x}{\in R}_{j})$ is the indicator function for the region $R$ defined as:

$$I\left( \vec{x}{\in R}_{j} \right)=\left\{ \begin{aligned} 1 (\vec{x}{\in R}_{j}) \\ 0 (\vec{x}\notin R_{j}) \end{aligned} \right.$$

The GBDT model includes $M$ decision trees with parameters $\vec{\theta}=(\vec{\theta_{1}},\ldots,\vec{\theta_{M}})$. Hence, the GBDT model is shown as follows:

$$g\left( \vec{x};\vec{\theta} \right)=\sum_{m=1}^{M} T(\vec{x};\vec{\theta_{m}})$$

$$f\left( \vec{x};\vec{\theta} \right)=\sigma(g(\vec{x};\vec{\theta}))$$

**Deep neural network (DNN)**

DNN is an automated machine learning approach with an open source, in-memory, rapid, and scalable machine learning and predictive analytics platform (<https://docs.h2o.ai/h2o/latest-stable/h2o-docs/index.html>). Many categorization or pattern recognition issues are handled by ANNs. They have been taught to produce an output by combining the input variables. This approach often involves the use of many hidden layers that mathematically reflect the brain connections. Even while ANNs are the industry standard for many categorization jobs, they have several limitations. Their standardized, layered design takes a lot of time and can produce extremely subpar results. This particular method is also referred to as a “black-box” technology. It is nearly impossible to determine how it accomplishes the categorization process or the reason an ANN did not function. The equation of the neuron’s processing process is deployed as below:

$$v_{a}=\sum_{j} w_{ab}x_{b}+h_{a}$$

$$y_{a}=f(v_{a})$$

Here, $w_{ab}$ stands for the connection weight of the input terminal, that is, the binding strength, and $y_{a}$ is the output signal. $h_{a}$ indicates the closed value, $v_{a}$ indicates the internal state of the neuron, $x_{b}$ symbolizes the input signal, and $f$ represents the activation function, and $j$ is the number of neurons.

**Supplementary contents of performance assessment**

**Brier score**

The Brier score is equal to the mean squared error between the actual outcome and the predicted probability (10), with lower values indicating better calibration and a value of more than 0.25 representing a useless model. The below equation is used to explain Brier score:

$$Brier Score= \frac{1}{N}\sum_{i=1}^{n} {(p_{i}-o_{i})}^{2}$$

Here, $N$ indicates the total number of enrolled individuals, $p_{i}$ represents the predicted probability, and $o_{i}$ represents the observed probability (“0” or “1”).

**Confusion matrix**

Specificity, sensitivity, negative predictive value, precision, and accuracy were deduced from confusion matrix. The Youden index is the sum of sensitivity and specificity, with a larger value indicating better performance of models.


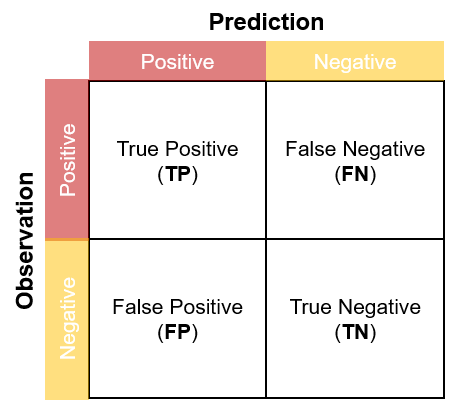


Specificity = $1-FP/(TN+FP)$

Sensitivity = $TP/(TP+FN)$

Negative predictive value= $TN/(TN+FN)$

Precision = $TP/(TP+FP)$

Accuracy =$(TP+TN)/(TP+FN+FP+TN)$

Youden index=specificity$+$sensitivity

**Supplementary contents of feature importance**

For analyzing feature importance, Shaley Additive exPlanation (SHAP) was utilized to interpret the feature contribution to outcome. We adopted the following formula:

$$g\left( z^{'} \right)=\phi_{0}+\sum_{j=1}^{M} \phi_{j}{Z^{'}}_{j}$$

Among them, $g$ represents the interpretation model, $M$ stands for the number of input parameters, $\phi_{0}$ is a constant, and $\phi_{j}$ indicates the attribution value (Shapley value) of each feature.

**Development of online calculator**

To begin with, the optimal model was saved as the format of PKL document via Python software (version 3.9.7). Next, the optimal model and corresponding Python code were both uploaded to the GitHub (<https://github.com/>) website. Finally, a web-based calculator was deployed after interlinking the Streamlit app (<https://share.streamlit.io/>) into the GitHub.

**Risk stratification system according to the optimal model**

Based on the threshold of the optimal model, participants were categorized into a low-risk group and a high-risk group. Participants with a predicted probability of threshold or below were classified into the low-risk group, whereas participants with a predicted probability of above threshold were classified in the high-risk group. In addition, a comparison of actual probability was conducted between the two risk groups.

**Results**

**Learning curves**





Learning curve for the logistic regression technique. A. Before random hyper-parameter search; B. After random hyper-parameter search. The x-axis is training set size, and the y-axis is area under curve. After random hyper-parameter search, the red line (training scores) become closer to the blue line (x-val scores).





Learning curve for the decision tree technique. A. Before random hyper-parameter search; B. After random hyper-parameter search. The x-axis is training set size, and the y-axis is area under curve. After random hyper-parameter search, the red line (training scores) become closer to the blue line (x-val scores).





Learning curve for the random forest technique. A. Before random hyper-parameter search; B. After random hyper-parameter search. The x-axis is training set size, and the y-axis is area under curve. After random hyper-parameter search, the red line (training scores) become closer to the blue line (x-val scores).





Learning curve for the support vector regression technique. A. Before random hyper-parameter search; B. After random hyper-parameter search. The x-axis is training set size, and the y-axis is area under curve. After random hyper-parameter search, the red line (training scores) become closer to the blue line (x-val scores).





Learning curve for the gradient boosting decision tree technique. A. Before random hyper-parameter search; B. After random hyper-parameter search. The x-axis is training set size, and the y-axis is area under curve. After random hyper-parameter search, the red line (training scores) become closer to the blue line (x-val scores).

**Area under the receiver operating curve in the external validation set**


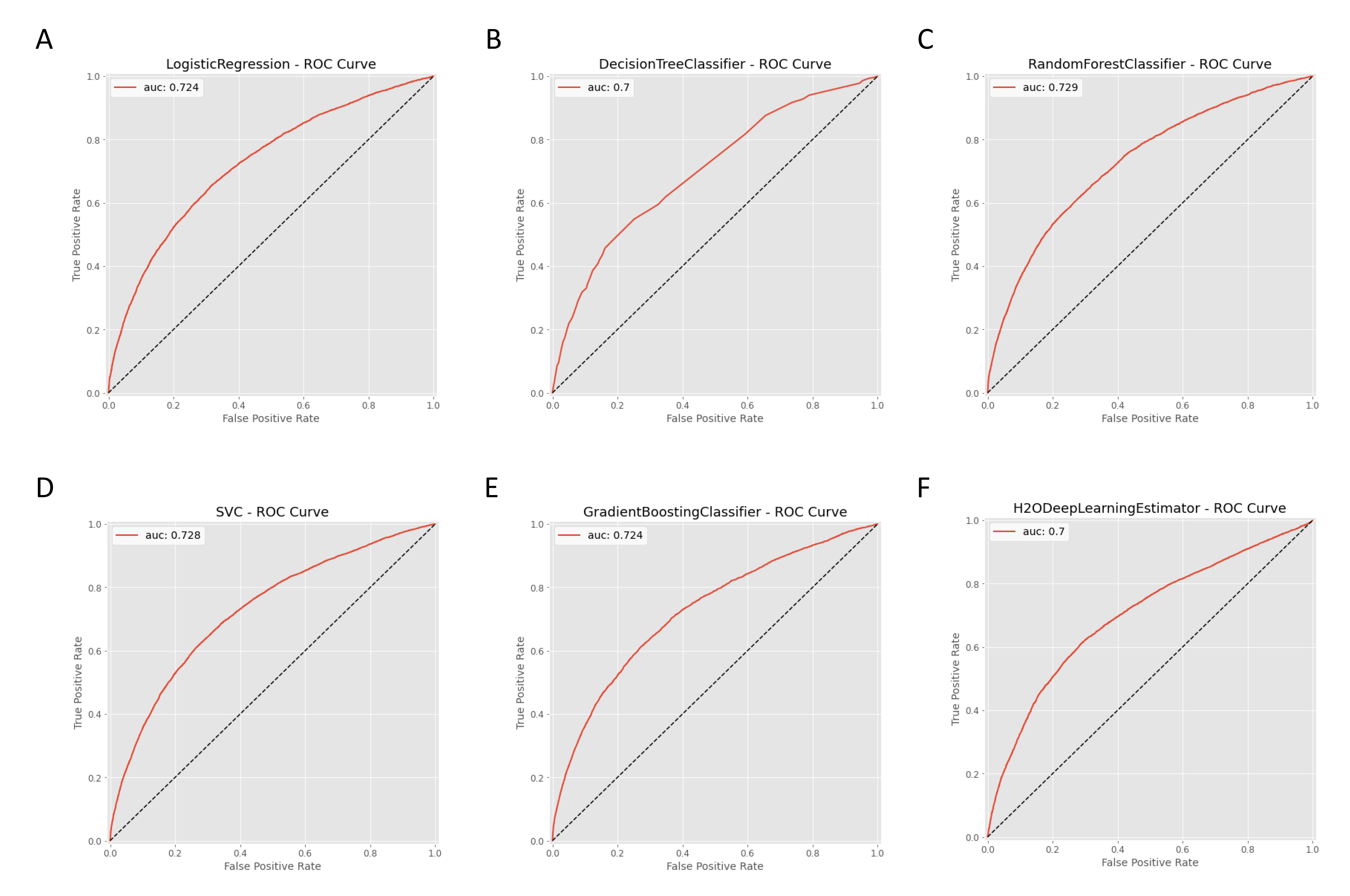


Area under the receiver operating curve for the six machine-learning models in the external validation set. A. Logistic Regression; B. Decision Tree; C. Random Forest; D. Support Vector Machine; E. Gradient Boosting Decision Tree; F. Deep Neural Network. The x-axis is false positive rate, and the y-axis is true positive rate. The dotted black line is random prediction.

**Violin plots for the discrimination slopes in the external validation set**


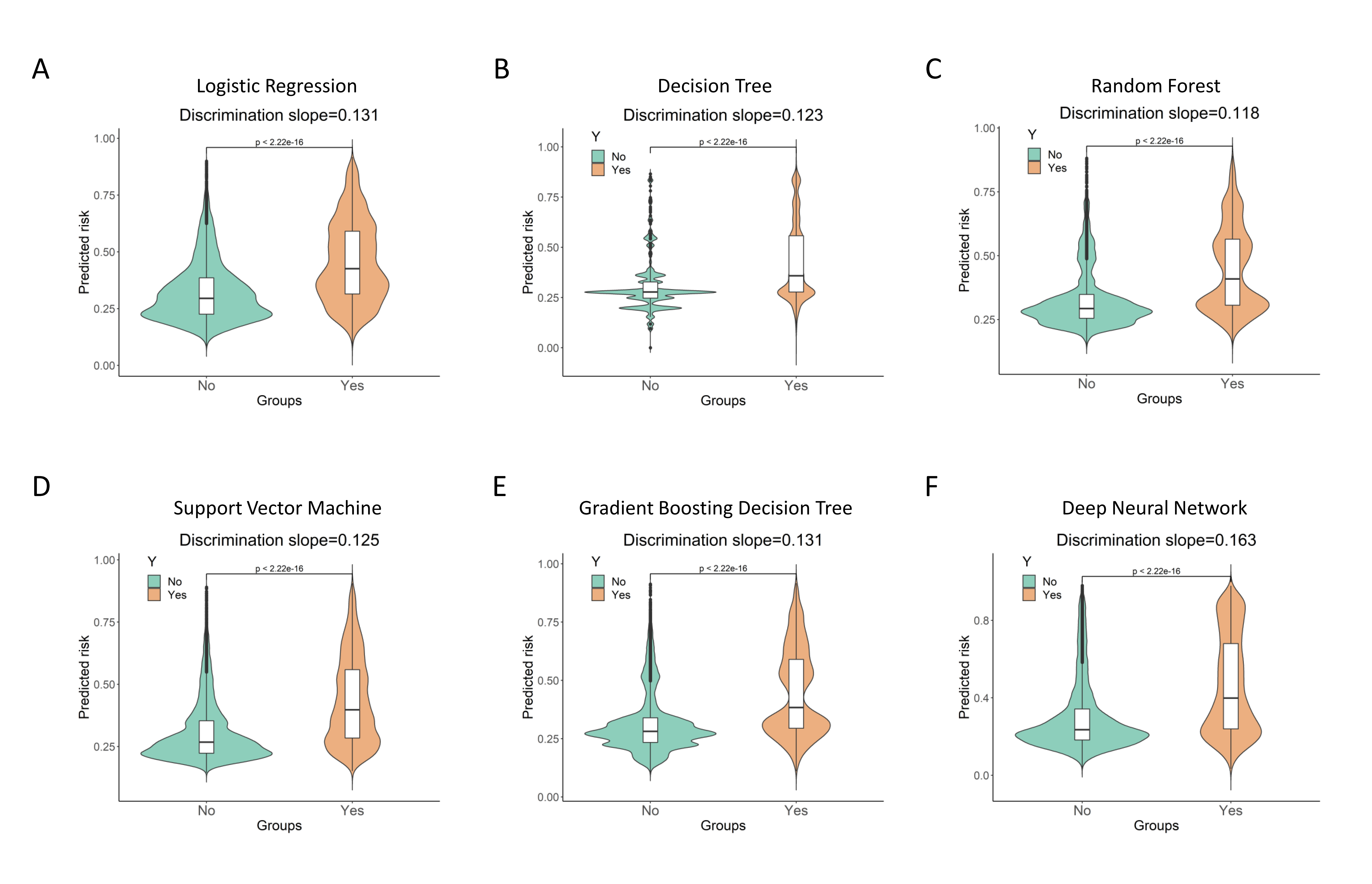


Violin plots for the discrimination slopes of the six machine-learning models in the external validation set. A. Logistic Regression; B. Decision Tree; C. Random Forest; D. Support Vector Machine; E. Gradient Boosting Decision Tree; F. Deep Neural Network. The discrimination slope is calculated as the difference between the mean predicted probability with and without medical disputes.

**Calibration curves in the external validation set**


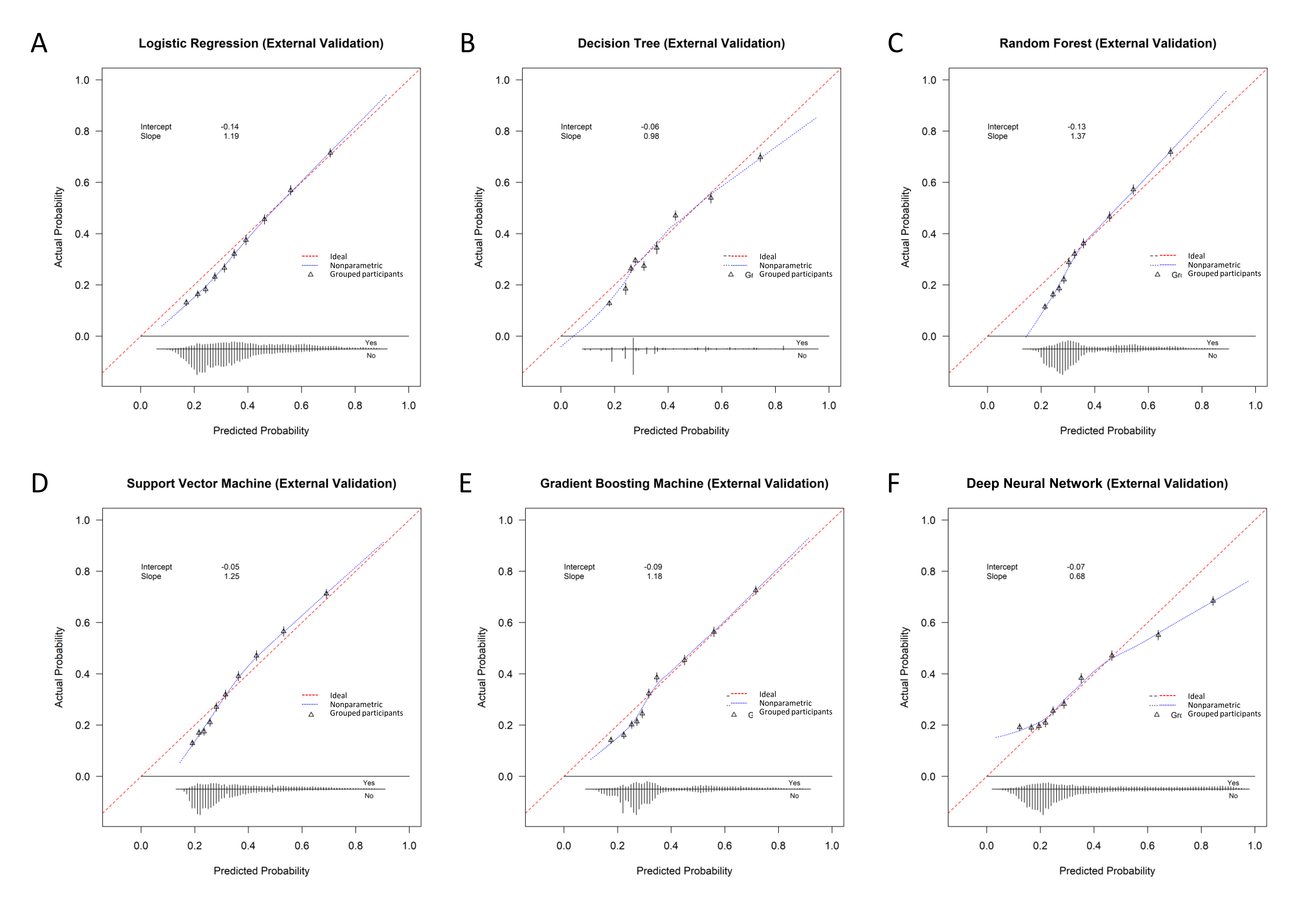


Calibration curves for the six machine-learning models in the external validation set. A. Logistic Regression; B. Decision Tree; C. Random Forest; D. Support Vector Machine; E. Gradient Boosting Decision Tree; F. Deep Neural Network. The x-axis is predicted probability, and the y-axis is actual probability. The dotted red line indicates perfect match of models. The closer the dotted blue line is to dotted red line, the better prediction performance the model has.

**Decision curve analysis in the external validation set**


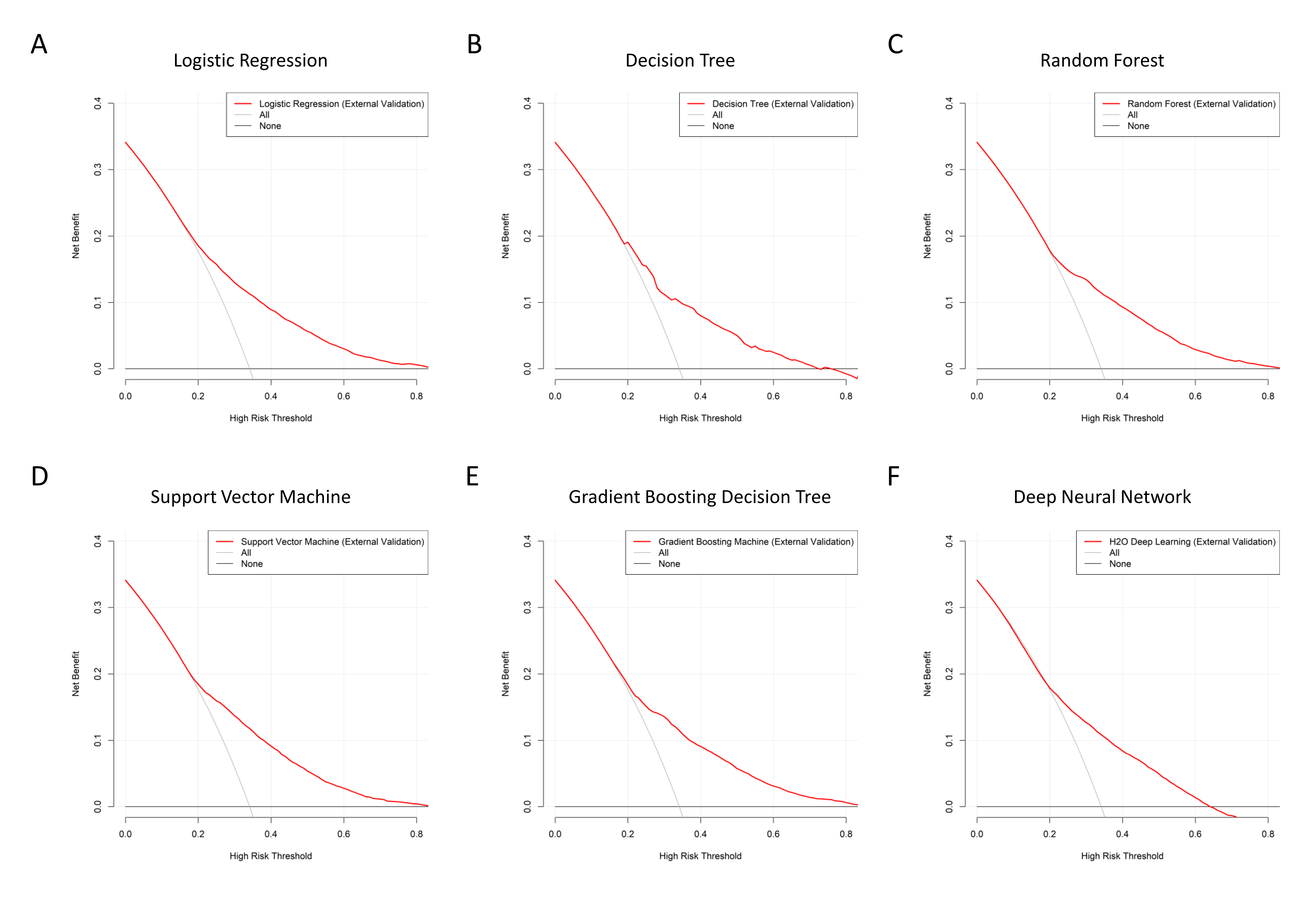


Decision curve analysis for the six machine-learning models in the external validation set. A. Logistic Regression; B. Decision Tree; C. Random Forest; D. Support Vector Machine; E. Gradient Boosting Decision Tree; F. Deep Neural Network. The x-ais denotes the risk threshold, and the y-axis indicates the net benefit. The grey line denotes that all patients exhibited early death, and the black line indicates that no patients exhibited medical disputes.

**A comparison of model explanation using LIME, SHAP, and online calculator**

When it comes to model explanation in machine learning, there are several techniques available such as LIME, SHAP, and online calculator. Each of these methods has its own advantages and disadvantages. However, after careful consideration, the online calculator was chosen as the preferred option in this study.

LIME (Local Interpretable Model-Agnostic Explanations) is a popular technique for model explanation. It provides local explanations by approximating the model’s behavior around a specific instance. One of the advantages of LIME is its model-agnostic nature, which means it can be used with any machine learning model. It also provides interpretability at the feature level, allowing users to understand the contribution of each feature in the model’s decision-making process. However, LIME has some limitations. It relies on sampling and perturbing the input data, which may not always capture the full complexity of the model. Additionally, it may not be suitable for high-dimensional data or complex models due to its reliance on local approximations.

SHAP (SHapley Additive exPlanations) is another popular method for model explanation. It is based on game theory and provides a unified framework for interpreting the output of any machine learning model. SHAP values represent the contribution of each feature towards the model’s prediction. One advantage of SHAP is its theoretical foundation, which ensures consistency and fairness in the interpretation. It also provides global explanations, allowing users to understand the overall behavior of the model. However, SHAP has some limitations as well. It can be computationally expensive, especially for large datasets or complex models. It may also be difficult to interpret for non-technical users due to its reliance on complex mathematical concepts.

The online calculator, on the other hand, offers a different approach to model explanation. It provides a user-friendly interface that allows users to interactively explore the model’s behavior and understand its decision-making process. One advantage of the online calculator is its simplicity and ease of use. It does not require any technical knowledge or expertise to interpret the model’s output. It also provides real-time explanations, allowing users to understand the model’s behavior as it is making predictions. Additionally, the online calculator can handle both local and global explanations, providing a comprehensive understanding of the model’s behavior.

The decision to choose the online calculator as the preferred option for model explanation was based on several factors. Firstly, its simplicity and user-friendly interface make it accessible to a wide range of users, including non-technical stakeholders. This ensures that the model’s output can be easily understood and trusted by all parties involved. Secondly, the real-time explanations provided by the online calculator allow for immediate feedback and insights, enabling quick decision-making and adjustments if necessary. Lastly, the online calculator’s ability to handle both local and global explanations provides a comprehensive understanding of the model’s behavior, allowing users to explore different scenarios and analyze the impact of various factors on the model’s predictions.

Therefore, while techniques like LIME and SHAP have their own advantages and limitations, the online calculator was chosen as the preferred option for model explanation due to its simplicity, user-friendliness, real-time explanations, and ability to handle both local and global explanations.

**Supplementary references**

1. The National Health Commission of China. Circular of the general office of the national health commission on further strengthening the legal construction of medical and health institutions. Available at <http://wsjkw.hebei.gov.cn/tzgg/360249.jhtml> (Accessed by 2023, June 4th). 2019.

2. Garnica-Caparros M, Memmert D. Understanding gender differences in professional European football through machine learning interpretability and match actions data. Sci Rep. 2021 May 24;11(1):10805. The authors declare no competing interests. Epub 20210524. doi:10.1038/s41598-021-90264-w. Cited in: Pubmed; PMID 34031518.

3. Kourou K, Exarchos TP, Exarchos KP, Karamouzis MV, Fotiadis DI. Machine learning applications in cancer prognosis and prediction. Comput Struct Biotechnol J. 2015;13:8-17. Epub 20141115. doi:10.1016/j.csbj.2014.11.005. Cited in: Pubmed; PMID 25750696.

4. Kaminski B, Jakubczyk M, Szufel P. A framework for sensitivity analysis of decision trees. Cent Eur J Oper Res. 2018;26(1):135-159. Epub 20170524. doi:10.1007/s10100-017-0479-6. Cited in: Pubmed; PMID 29375266.

5. Wang B, He Z, Yi Z, Yuan C, Suo W, Pei S, Li Y, Ma H, Wang H, Xu B, Guo W, Huang X. Application of a decision tree model in the early identification of severe patients with severe fever with thrombocytopenia syndrome. PLoS One. 2021;16(7):e0255033. The authors have declared that no competing interests exist. Epub 20210730. doi:10.1371/journal.pone.0255033. Cited in: Pubmed; PMID 34329338.

6. Cutler DR, Edwards TC, Jr., Beard KH, Cutler A, Hess KT, Gibson J, Lawler JJ. Random forests for classification in ecology. Ecology. 2007 Nov;88(11):2783-92. doi:10.1890/07-0539.1. Cited in: Pubmed; PMID 18051647.

7. Ancuceanu R, Dinu M, Neaga I, Laszlo FG, Boda D. Development of QSAR machine learning-based models to forecast the effect of substances on malignant melanoma cells. Oncol Lett. 2019 May;17(5):4188-4196. Epub 20190225. doi:10.3892/ol.2019.10068. Cited in: Pubmed; PMID 31007759.

8. Chen HY, Chen JQ, Li JY, Huang HJ, Chen X, Zhang HY, Chen CY. Deep Learning and Random Forest Approach for Finding the Optimal Traditional Chinese Medicine Formula for Treatment of Alzheimer's Disease. J Chem Inf Model. 2019 Apr 22;59(4):1605-1623. Epub 20190402. doi:10.1021/acs.jcim.9b00041. Cited in: Pubmed; PMID 30888812.

9. Seto H, Oyama A, Kitora S, Toki H, Yamamoto R, Kotoku J, Haga A, Shinzawa M, Yamakawa M, Fukui S, Moriyama T. Gradient boosting decision tree becomes more reliable than logistic regression in predicting probability for diabetes with big data. Sci Rep. 2022 Oct 11;12(1):15889. The authors declare no competing interests. Epub 20221011. doi:10.1038/s41598-022-20149-z. Cited in: Pubmed; PMID 36220875.

10. Huang Y, Li W, Macheret F, Gabriel RA, Ohno-Machado L. A tutorial on calibration measurements and calibration models for clinical prediction models. J Am Med Inform Assoc. 2020 Apr 1;27(4):621-633. doi:10.1093/jamia/ocz228. Cited in: Pubmed; PMID 32106284.
