# Supplementary material for: Prediction of Medical Disputes Between Health Care Workers and Patients in Terms of Hospital Legal Construction Using Machine Learning Techniques: Externally Validated Cross-Sectional Study
Source: J Med Internet Res. 2023 Aug 17;25:e46854. doi: 10.2196/46854 (PMC10472173; doi:10.2196/46854)
Supplement: Multimedia Appendix 2 [file jmir_v25i1e46854_app2.docx]

| **Supplementary Table S1**. A comparison of characteristics between medical workers with and without medical dispute. | | | | |
| --- | --- | --- | --- | --- |
| Characteristics | Overall | Medical dispute | | P value |
|  |  | No | Yes |  |
| n | 38053 | 20526 | 17527 |  |
| Hospital type (Public/Private, %) | 36122/1931 (94.9/5.1) | 19363/1163 (94.3/5.7) | 16759/768 (95.6/4.4) | <0.001 |
| Hospital category (%) |  |  |  | <0.001 |
| General | 24189 (63.6) | 12603 (61.4) | 11586 (66.1) |  |
| Traditional Chinese Medicine | 7005 (18.4) | 4018 (19.6) | 2987 (17.0) |  |
| Integrated Chinese and western medicine | 1092 (2.9) | 636 (3.1) | 456 (2.6) |  |
| National | 47 (0.1) | 27 (0.1) | 20 (0.1) |  |
| Specialized | 3277 (8.6) | 1833 (8.9) | 1444 (8.2) |  |
| Maternity and child healthcare hospital | 2368 (6.2) | 1355 (6.6) | 1013 (5.8) |  |
| Others | 75 (0.2) | 54 (0.3) | 21 (0.1) |  |
| Tertiary hospital level (%) |  |  |  | 0.006 |
| Class A | 21642 (56.9) | 11579 (56.4) | 10063 (57.4) |  |
| Class B | 5242 (13.8) | 2932 (14.3) | 2310 (13.2) |  |
| Others | 11169 (29.4) | 6015 (29.3) | 5154 (29.4) |  |
| Occupation |  |  |  | <0.001 |
| Doctor | 14977 (39.4) | 6188 (30.1) | 8789 (50.1) |  |
| Pharmacist | 3042 (8.0) | 2089 (10.2) | 953 (5.4) |  |
| Nurse | 15843 (41.6) | 9347 (45.5) | 6496 (37.1) |  |
| Medical technicians | 4191 (11.0) | 2902 (14.1) | 1289 (7.4) |  |
| Technical title |  |  |  | <0.001 |
| Senior | 1411 (3.7) | 306 (1.5) | 1105 (6.3) |  |
| Vice senior | 6315 (16.6) | 2138 (10.4) | 4177 (23.8) |  |
| Middle | 15308 (40.2) | 8197 (39.9) | 7111 (40.6) |  |
| Junior | 13440 (35.3) | 8769 (42.7) | 4671 (26.7) |  |
| None | 1579 (4.1) | 1116 (5.4) | 463 (2.6) |  |
| Sex (Male/Female, %) | 11024/27029 (29.0/71.0) | 4432/16094 (21.6/78.4) | 6592/10935 (37.6/62.4) | <0.001 |
| Age |  |  |  | <0.001 |
| Below 30 years | 10624 (27.9) | 7103 (34.6) | 3521 (20.1) |  |
| 30-39 years | 17044 (44.8) | 9380 (45.7) | 7664 (43.7) |  |
| 40-49 years | 7927 (20.8) | 3136 (15.3) | 4791 (27.3) |  |
| 50 years and above | 2458 (6.5) | 907 (4.4) | 1551 (8.8) |  |
| Establishment of hospital legal construction |  |  |  | <0.001 |
| Yes | 36443 (95.8) | 19512 (95.1) | 16931 (96.6) |  |
| No | 132 (0.3) | 74 (0.4) | 58 (0.3) |  |
| Not clear | 1478 (3.9) | 940 (4.6) | 538 (3.1) |  |
| Understanding hospital president responsibility system under the leadership of the hospital Party committee |  |  |  | <0.001 |
| Very clear | 18235 (47.9) | 9030 (44.0) | 9205 (52.5) |  |
| Clear | 17129 (45.0) | 9784 (47.7) | 7345 (41.9) |  |
| Not clear | 2501 (6.6) | 1603 (7.8) | 898 (5.1) |  |
| Not concerned | 188 (0.5) | 109 (0.5) | 79 (0.5) |  |
| Independent rule of law department in the hospital |  |  |  | <0.001 |
| Yes | 27093 (71.2) | 14153 (69.0) | 12940 (73.8) |  |
| No | 3102 (8.2) | 1651 (8.0) | 1451 (8.3) |  |
| Not clear | 7858 (20.7) | 4722 (23.0) | 3136 (17.9) |  |
| Understanding the duty of hospital law department |  |  |  | <0.001 |
| Very clear | 12347 (32.4) | 6212 (30.3) | 6135 (35.0) |  |
| Clear | 19206 (50.5) | 10446 (50.9) | 8760 (50.0) |  |
| Not clear | 6228 (16.4) | 3714 (18.1) | 2514 (14.3) |  |
| Not concerned | 272 (0.7) | 154 (0.8) | 118 (0.7) |  |
| Understanding the duty of legal counselor in the hospital |  |  |  | <0.001 |
| Very clear | 10812 (28.4) | 5414 (26.4) | 5398 (30.8) |  |
| Clear | 17560 (46.1) | 9416 (45.9) | 8144 (46.5) |  |
| Not clear | 9194 (24.2) | 5401 (26.3) | 3793 (21.6) |  |
| None | 194 (0.5) | 117 (0.6) | 77 (0.4) |  |
| Not concerned | 293 (0.8) | 178 (0.9) | 115 (0.7) |  |
| Understanding the contents of hospital charters |  |  |  | <0.001 |
| Very clear | 15577 (40.9) | 8018 (39.1) | 7559 (43.1) |  |
| Clear | 19150 (50.3) | 10637 (51.8) | 8513 (48.6) |  |
| Not clear | 3153 (8.3) | 1773 (8.6) | 1380 (7.9) |  |
| None | 42 (0.1) | 22 (0.1) | 20 (0.1) |  |
| Not concerned | 131 (0.3) | 76 (0.4) | 55 (0.3) |  |
| Hospital performance appraisal system including hospital legal construction |  |  |  | <0.001 |
| Yes | 29190 (76.7) | 15403 (75.0) | 13787 (78.7) |  |
| No | 1002 (2.6) | 518 (2.5) | 484 (2.8) |  |
| Not clear | 7098 (18.7) | 4173 (20.3) | 2925 (16.7) |  |
| No performance appraisal system | 548 (1.4) | 309 (1.5) | 239 (1.4) |  |
| Not concerned | 215 (0.6) | 123 (0.6) | 92 (0.5) |  |
| Importance of clinical practice in accordance with law |  |  |  | 0.003 |
| Very important | 31905 (83.8) | 17121 (83.4) | 14784 (84.3) |  |
| Important | 5219 (13.7) | 2868 (14.0) | 2351 (13.4) |  |
| Slightly important | 871 (2.3) | 513 (2.5) | 358 (2.0) |  |
| Not important | 39 (0.1) | 17 (0.1) | 22 (0.1) |  |
| Very not important | 19 (0.0) | 7 (0.0) | 12 (0.1) |  |
| Rule of law training for new recruits (No/Yes, %) | 6979/31074 (18.3/81.7) | 3872/16654 (18.9/81.1) | 3107/14420 (17.7/82.3) | 0.004 |
| Examination of law popularization among hospital staffs (No/Yes, %) | 5104/32949 (13.4/86.6) | 2894/17632 (14.1/85.9) | 2210/15317 (12.6/87.4) | <0.001 |
| Legal training organized by hospital (No/Yes, %) | 2338/35715 (6.1/93.9) | 1177/19349 (5.7/94.3) | 1161/16366 (6.6/93.4) | <0.001 |
| Publicity of rule of law for hospital staffs (No/Yes, %) | 2792/35261 (7.3/92.7) | 1507/19019 (7.3/92.7) | 1285/16242 (7.3/92.7) | 0.985 |
| Publicity of rule of law for patient’s family members (No/Yes, %) | 10693/27360 (28.1/71.9) | 5920/14606 (28.8/71.2) | 4773/12754 (27.2/72.8) | 0.001 |
| Participating in legal training organized by hospitals (No/Yes, %) | 6791/31262 (17.8/82.2) | 3609/16917 (17.6/82.4) | 3182/14345 (18.2/81.8) | 0.15 |
| Necessity of carrying out hospital legal training |  |  |  | <0.001 |
| Very necessary | 28857 (75.8) | 15169 (73.9) | 13688 (78.1) |  |
| Necessary | 8049 (21.2) | 4661 (22.7) | 3388 (19.3) |  |
| Slightly necessary | 995 (2.6) | 609 (3.0) | 386 (2.2) |  |
| Unnecessary | 119 (0.3) | 68 (0.3) | 51 (0.3) |  |
| Very unnecessary | 33 (0.1) | 19 (0.1) | 14 (0.1) |  |
| Willingness to participate in law training organized by the hospital |  |  |  | <0.001 |
| Very willing | 26360 (69.3) | 13986 (68.1) | 12374 (70.6) |  |
| Willing | 8964 (23.6) | 4947 (24.1) | 4017 (22.9) |  |
| Slightly willing | 2427 (6.4) | 1422 (6.9) | 1005 (5.7) |  |
| Unwilling | 220 (0.6) | 118 (0.6) | 102 (0.6) |  |
| Very unwilling | 82 (0.2) | 53 (0.3) | 29 (0.2) |  |
| Helpfulness of the hospital’s legal construction to your medical work |  |  |  | <0.001 |
| Very helpful | 26819 (70.5) | 14119 (68.8) | 12700 (72.5) |  |
| Helpful | 9199 (24.2) | 5190 (25.3) | 4009 (22.9) |  |
| Slightly helpful | 1891 (5.0) | 1139 (5.5) | 752 (4.3) |  |
| Unhelpful | 132 (0.3) | 72 (0.4) | 60 (0.3) |  |
| Very unhelpful | 12 (0.0) | 6 (0.0) | 6 (0.0) |  |
| Previously facing legal issue outside of medical works (No/Yes, %) | 18070/19983 (47.5/52.5) | 12664/7862 (61.7/38.3) | 5406/12121 (30.8/69.2) | <0.001 |
| Construction status of hospital rule of law |  |  |  | 0.125 |
| Very good | 15108 (39.7) | 8233 (40.1) | 6875 (39.2) |  |
| Good | 17019 (44.7) | 9183 (44.7) | 7836 (44.7) |  |
| Slightly good | 5665 (14.9) | 2977 (14.5) | 2688 (15.3) |  |
| Bad | 201 (0.5) | 102 (0.5) | 99 (0.6) |  |
| Very bad | 60 (0.2) | 31 (0.2) | 29 (0.2) |  |

| **Supplementary Table S2**. Multivariate analysis of characteristics for predicting medical disputes among medical workers in the training group. | | | | |
| --- | --- | --- | --- | --- |
| Characteristics | OR | 95% CI | | P value |
|  |  | LL | UL |  |
| (Intercept) | 2.02 | 1.93 | 2.11 | <0.001 |
| Hospital type |  |  |  |  |
| Public | Reference |  |  |  |
| Private | 0.97 | 0.94 | 0.99 | 0.006 |
| Hospital category |  |  |  |  |
| General | Reference |  |  |  |
| Traditional Chinese Medicine | 0.96 | 0.94 | 0.97 | <0.001 |
| Integrated Chinese and western medicine | 0.95 | 0.92 | 0.98 | 0.001 |
| National | 0.93 | 0.81 | 1.06 | 0.257 |
| Specialized | 0.97 | 0.95 | 0.99 | <0.001 |
| Maternity and child healthcare hospital | 0.97 | 0.95 | 0.99 | 0.005 |
| Others | 0.90 | 0.81 | 1.00 | 0.049 |
| Tertiary hospital level |  |  |  |  |
| Class A | Reference |  |  |  |
| Class B | 0.99 | 0.97 | 1.00 | 0.126 |
| Others | 0.98 | 0.97 | 1.00 | 0.007 |
| Occupation |  |  |  |  |
| Doctor | Reference |  |  |  |
| Pharmacist | 0.84 | 0.82 | 0.85 | <0.001 |
| Nurse | 0.98 | 0.97 | 0.99 | 0.001 |
| Medical technicians | 0.83 | 0.82 | 0.85 | <0.001 |
| Technical title |  |  |  |  |
| Senior | Reference |  |  |  |
| Vice senior | 0.94 | 0.91 | 0.96 | <0.001 |
| Middle | 0.84 | 0.81 | 0.86 | <0.001 |
| Junior | 0.80 | 0.77 | 0.82 | <0.001 |
| None | 0.79 | 0.76 | 0.82 | <0.001 |
| Sex |  |  |  |  |
| Male | Reference |  |  |  |
| Female | 0.90 | 0.89 | 0.91 | <0.001 |
| Age |  |  |  |  |
| Below 30 years | Reference |  |  |  |
| 30-39 years | 1.03 | 1.01 | 1.04 | 0.001 |
| 40-49 years | 1.10 | 1.08 | 1.12 | <0.001 |
| 50 years and above | 1.11 | 1.09 | 1.14 | <0.001 |
| Establishment of hospital legal construction |  |  |  |  |
| Yes | Reference |  |  |  |
| No | 0.97 | 0.90 | 1.06 | 0.542 |
| Not clear | 0.99 | 0.96 | 1.01 | 0.287 |
| Understanding hospital president responsibility system under the leadership of the hospital Party committee |  |  |  |  |
| Very clear | Reference |  |  |  |
| Clear | 0.99 | 0.98 | 1.01 | 0.243 |
| Not clear | 0.98 | 0.95 | 1.00 | 0.093 |
| Not concerned | 1.02 | 0.94 | 1.10 | 0.647 |
| Independent rule of law department in the hospital |  |  |  |  |
| Yes | Reference |  |  |  |
| No | 0.99 | 0.97 | 1.01 | 0.175 |
| Not clear | 0.99 | 0.97 | 1.00 | 0.072 |
| Understanding the duty of hospital law department |  |  |  |  |
| Very clear | Reference |  |  |  |
| Clear | 1.02 | 1.00 | 1.04 | 0.072 |
| Not clear | 1.02 | 0.99 | 1.05 | 0.228 |
| Not concerned | 1.09 | 1.01 | 1.17 | 0.024 |
| Understanding the duty of legal counselor in the hospital | Reference |  |  |  |
| Very clear |  |  |  |  |
| Clear | 0.99 | 0.97 | 1.02 | 0.509 |
| Not clear | 1.01 | 0.98 | 1.04 | 0.734 |
| None | 0.98 | 0.91 | 1.06 | 0.649 |
| Not concerned | 1.08 | 1.00 | 1.17 | 0.045 |
| Understanding the contents of hospital charters |  |  |  |  |
| Very clear | Reference |  |  |  |
| Clear | 1.01 | 1.00 | 1.03 | 0.134 |
| Not clear | 1.06 | 1.04 | 1.09 | <0.001 |
| None | 0.97 | 0.84 | 1.13 | 0.698 |
| Not concerned | 0.98 | 0.89 | 1.08 | 0.743 |
| Hospital performance appraisal system including hospital legal construction |  |  |  |  |
| Yes | Reference |  |  |  |
| No | 1.00 | 0.97 | 1.04 | 0.766 |
| Not clear | 1.00 | 0.98 | 1.01 | 0.905 |
| No performance appraisal system | 1.01 | 0.97 | 1.05 | 0.690 |
| Not concerned | 0.99 | 0.92 | 1.06 | 0.759 |
| Importance of clinical practice in accordance with law |  |  |  |  |
| Very important | Reference |  |  |  |
| Important | 1.03 | 1.01 | 1.04 | 0.001 |
| Slightly important | 1.02 | 0.99 | 1.06 | 0.190 |
| Not important | 1.11 | 0.96 | 1.29 | 0.157 |
| Very not important | 1.35 | 1.08 | 1.69 | 0.008 |
| Rule of law training for new recruits |  |  |  |  |
| No | Reference |  |  |  |
| Yes | 1.01 | 1.00 | 1.03 | 0.051 |
| Examination of law popularization among hospital staffs |  |  |  |  |
| No | Reference |  |  |  |
| Yes | 1.00 | 0.98 | 1.01 | 0.587 |
| Legal training organized by hospitals |  |  |  |  |
| No | Reference |  |  |  |
| Yes | 0.96 | 0.94 | 0.98 | <0.001 |
| Publicity of rule of law for hospital staffs |  |  |  |  |
| No | Reference |  |  |  |
| Yes | 0.97 | 0.95 | 0.99 | 0.014 |
| Publicity of rule of law for patient’s family members |  |  |  |  |
| No | Reference |  |  |  |
| Yes | 1.01 | 0.99 | 1.02 | 0.374 |
| Participating in legal training organized by hospitals |  |  |  |  |
| No | Reference |  |  |  |
| Yes | 0.97 | 0.95 | 0.98 | <0.001 |
| Necessity of carrying out hospital legal training |  |  |  |  |
| Very necessary | Reference |  |  |  |
| Necessary | 0.97 | 0.96 | 0.99 | 0.001 |
| Slightly necessary | 0.95 | 0.91 | 0.99 | 0.007 |
| Unnecessary | 1.01 | 0.92 | 1.11 | 0.755 |
| Very unnecessary | 0.92 | 0.78 | 1.09 | 0.353 |
| Willingness to participate in law training organized by hospitals |  |  |  |  |
| Very willing | Reference |  |  |  |
| Willing | 1.03 | 1.01 | 1.05 | <0.001 |
| Slightly willing | 1.02 | 0.99 | 1.05 | 0.199 |
| Unwilling | 1.06 | 0.99 | 1.15 | 0.105 |
| Very unwilling | 0.96 | 0.86 | 1.07 | 0.427 |
| Helpfulness of the hospital’s legal construction to your medical work |  |  |  |  |
| Very helpful | Reference |  |  |  |
| Helpful | 0.98 | 0.97 | 1.00 | 0.076 |
| Slightly helpful | 0.99 | 0.96 | 1.02 | 0.476 |
| Unhelpful | 1.08 | 0.98 | 1.18 | 0.115 |
| Very unhelpful | 1.06 | 0.82 | 1.38 | 0.659 |
| Previously facing legal issue outside of medical works |  |  |  |  |
| No | Reference |  |  |  |
| Yes | 1.28 | 1.27 | 1.29 | <0.001 |
| Construction status of hospital rule of law |  |  |  |  |
| Very good | Reference |  |  |  |
| Good | 1.05 | 1.03 | 1.06 | <0.001 |
| Slightly good | 1.09 | 1.07 | 1.12 | <0.001 |
| Bad | 1.10 | 1.03 | 1.18 | 0.006 |
| Very bad | 0.96 | 0.84 | 1.09 | 0.490 |
| OR, odds ratio; CI, confident interval; LL, lower limit; UL, upper limit. | | | | |

| **Supplementary Table S3**. Machine learning-based approaches and their full hyperparameters. | |
| --- | --- |
| Approaches | Hyperparameters |
| Logistic regression | LogisticRegression(C=0.01, random_state=42) |
| Decision tree | DecisionTreeClassifier(max_depth=7, max_features='log2', min_samples_leaf=9, min_samples_split=176, random_state=42) |
| Random forest | RandomForestClassifier(max_depth=97, max_features='log2', min_samples_leaf=54, min_samples_split=61, n_estimators=44, random_state=42) |
| Gradient boosting machine | GradientBoostingClassifier(max_depth=2, max_features='auto', min_samples_leaf=49, min_samples_split=141, n_estimators=148, random_state=42) |
| Support vector classification | SVC (C=0.016599452033620267, gamma=0.02904180608409973, probability=True, kernel=rbf) |

| **Supplementary Table S4**. Baseline clinical characteristics of medical workers from the external validation group and a comparison between participants with and without medical disputes. | | | | |
| --- | --- | --- | --- | --- |
| Characteristics | Overall | Medical disputes | | P |
|  |  | No | Yes |  |
| n | 26285 | 17318 | 8967 |  |
| Hospital type (Public/Private, %) | 24455/1830 (93.0/7.0) | 15988/1330 (92.3/7.7) | 8467/500 (94.4/5.6) | <0.001 |
| Hospital category (%) |  |  |  | 0.023 |
| General | 12964 (49.3) | 8459 (48.8) | 4505 (50.2) |  |
| Traditional Chinese Medicine | 3915 (14.9) | 2556 (14.8) | 1359 (15.2) |  |
| Integrated Chinese and western medicine | 3055 (11.6) | 2057 (11.9) | 998 (11.1) |  |
| National | 8 (0.0) | 7 (0.0) | 1 (0.0) |  |
| Specialized | 5472 (20.8) | 3663 (21.2) | 1809 (20.2) |  |
| Maternity and child healthcare hospital | 710 (2.7) | 458 (2.6) | 252 (2.8) |  |
| Others | 161 (0.6) | 118 (0.7) | 43 (0.5) |  |
| Tertiary hospital level (%) |  |  |  | 0.02 |
| Class A | 16382 (62.3) | 10702 (61.8) | 5680 (63.3) |  |
| Class B | 2158 (8.2) | 1468 (8.5) | 690 (7.7) |  |
| Others | 7745 (29.5) | 5148 (29.7) | 2597 (29.0) |  |
| Occupation |  |  |  | <0.001 |
| Doctor | 8899 (33.9) | 4498 (26.0) | 4401 (49.1) |  |
| Pharmacist | 1846 (7.0) | 1328 (7.7) | 518 (5.8) |  |
| Nurse | 13184 (50.2) | 9640 (55.7) | 3544 (39.5) |  |
| Medical technicians | 2356 (9.0) | 1852 (10.7) | 504 (5.6) |  |
| Technical title |  |  |  | <0.001 |
| Senior | 1256 (4.8) | 362 (2.1) | 894 (10.0) |  |
| Vice senior | 2703 (10.3) | 1086 (6.3) | 1617 (18.0) |  |
| Middle | 10512 (40.0) | 6514 (37.6) | 3998 (44.6) |  |
| Junior | 10473 (39.8) | 8211 (47.4) | 2262 (25.2) |  |
| None | 1341 (5.1) | 1145 (6.6) | 196 (2.2) |  |
| Sex (Male/Female, %) | 5499/20786 (20.9/79.1) | 2994/14324 (17.3/82.7) | 2505/6462 (27.9/72.1) | <0.001 |
| Age |  |  |  | <0.001 |
| Below 30 years | 6361 (24.2) | 5299 (30.6) | 1062 (11.8) |  |
| 30-39 years | 10899 (41.5) | 7464 (43.1) | 3435 (38.3) |  |
| 40-49 years | 5832 (22.2) | 3046 (17.6) | 2786 (31.1) |  |
| 50 years and above | 3193 (12.1) | 1509 (8.7) | 1684 (18.8) |  |
| Understanding the duty of hospital law department |  |  |  | <0.001 |
| Very clear | 3907 (14.9) | 2859 (16.5) | 1048 (11.7) |  |
| Clear | 10835 (41.2) | 7375 (42.6) | 3460 (38.6) |  |
| Not clear | 9609 (36.6) | 6054 (35.0) | 3555 (39.6) |  |
| Not concerned | 1393 (5.3) | 785 (4.5) | 608 (6.8) |  |
| None | 541 (2.1) | 245 (1.4) | 296 (3.3) |  |
| Understanding the duty of legal counselor in the hospital |  |  |  | <0.001 |
| Very clear | 2701 (10.3) | 2030 (11.7) | 671 (7.5) |  |
| Clear | 6690 (25.5) | 4468 (25.8) | 2222 (24.8) |  |
| Not clear | 11492 (43.7) | 7409 (42.8) | 4083 (45.5) |  |
| Not concerned | 5249 (20.0) | 3329 (19.2) | 1920 (21.4) |  |
| None | 153 (0.6) | 82 (0.5) | 71 (0.8) |  |
| Understanding the contents of hospital charters |  |  |  | <0.001 |
| Very clear | 4854 (18.5) | 3566 (20.6) | 1288 (14.4) |  |
| Clear | 14369 (54.7) | 9409 (54.3) | 4960 (55.3) |  |
| Not clear | 5991 (22.8) | 3732 (21.5) | 2259 (25.2) |  |
| Not concerned | 1022 (3.9) | 590 (3.4) | 432 (4.8) |  |
| None | 49 (0.2) | 21 (0.1) | 28 (0.3) |  |
| Importance of clinical practice in accordance with law |  |  |  | 0.009 |
| Very important | 20154 (76.7) | 13368 (77.2) | 6786 (75.7) |  |
| Important | 5451 (20.7) | 3503 (20.2) | 1948 (21.7) |  |
| Slightly important | 639 (2.4) | 426 (2.5) | 213 (2.4) |  |
| Not important | 20 (0.1) | 12 (0.1) | 8 (0.1) |  |
| Very not important | 21 (0.1) | 9 (0.1) | 12 (0.1) |  |
| Legal training organized by hospital (No/Yes, %) | 2813/23472 (10.7/89.3) | 1916/15402 (11.1/88.9) | 897/8070 (10.0/90.0) | 0.009 |
| Publicity of rule of law for hospital staffs (No/Yes, %) | 1294/24991 (4.9/95.1) | 811/16507 (4.7/95.3) | 483/8484 (5.4/94.6) | 0.014 |
| Participating in legal training organized by hospitals (No/Yes, %) | 1869/24416 (7.1/92.9) | 1195/16123 (6.9/93.1) | 674/8293 (7.5/92.5) | 0.069 |
| Necessity of carrying out hospital legal training |  |  |  | <0.001 |
| Very necessary | 17384 (66.1) | 11627 (67.1) | 5757 (64.2) |  |
| Necessary | 7545 (28.7) | 4874 (28.1) | 2671 (29.8) |  |
| Slightly necessary | 1241 (4.7) | 768 (4.4) | 473 (5.3) |  |
| Unnecessary | 66 (0.3) | 29 (0.2) | 37 (0.4) |  |
| Very unnecessary | 49 (0.2) | 20 (0.1) | 29 (0.3) |  |
| Willingness to participate in law training organized by the hospital |  |  |  | <0.001 |
| Very willing | 14384 (54.7) | 9946 (57.4) | 4438 (49.5) |  |
| Willing | 9406 (35.8) | 5957 (34.4) | 3449 (38.5) |  |
| Slightly willing | 2269 (8.6) | 1303 (7.5) | 966 (10.8) |  |
| Unwilling | 147 (0.6) | 79 (0.5) | 68 (0.8) |  |
| Very unwilling | 79 (0.3) | 33 (0.2) | 46 (0.5) |  |
| Previously facing legal issue outside of medical work (No/Yes, %) | 23174/3111 (88.2/11.8) | 16169/1149 (93.4/6.6) | 7005/1962 (78.1/21.9) | <0.001 |
| Construction status of hospital rule of law |  |  |  | <0.001 |
| Very good | 8594 (32.7) | 6511 (37.6) | 2083 (23.2) |  |
| Good | 10654 (40.5) | 7062 (40.8) | 3592 (40.1) |  |
| Slightly good | 6616 (25.2) | 3568 (20.6) | 3048 (34.0) |  |
| Bad | 279 (1.1) | 120 (0.7) | 159 (1.8) |  |
| Very bad | 142 (0.5) | 57 (0.3) | 85 (0.9) |  |

| **Supplementary Table S5.** Prediction performance of machine learning approaches for predicting medical disputes among medical workers in the external validation group. | | | | | | |
| --- | --- | --- | --- | --- | --- | --- |
| Measures | Approaches | | | | | |
|  | Logistic regression | Decision tree | Random forest | SVC | Gradient boosting machine | H2O deep learning |
| Mean actual (Validation group) | 34.12% | 34.12% | 34.12% | 34.12% | 34.12% | 34.12% |
| Mean predicted | 36.87% | 35.32% | 36.78% | 35.07% | 36.02% | 35.41% |
| Brier score | 0.193 | 0.198 | 0.193 | 0.192 | 0.192 | 0.201 |
| Intercept | -0.135 | -0.061 | -0.128 | -0.047 | -0.095 | -0.073 |
| Calibration slope | 1.190 | 0.978 | 1.373 | 1.252 | 1.178 | 0.677 |
| AUC (95%CI) | 0.724 (0.718-0.731) | 0.700 (0.693-0.706) | 0.729 (0.722-0.735) | 0.728 (0.721-0.734) | 0.724 (0.718- 0.731) | 0.700 (0.693-0.707) |
| Discrimination slope | 0.131 | 0.123 | 0.118 | 0.125 | 0.131 | 0.163 |
| Specificity | 0.686 | 0.749 | 0.721 | 0.733 | 0.727 | 0.710 |
| Sensitivity | 0.652 | 0.549 | 0.615 | 0.612 | 0.613 | 0.613 |
| NPV | 0.792 | 0.762 | 0.783 | 0.785 | 0.784 | 0.780 |
| Precision (PPV) | 0.518 | 0.531 | 0.533 | 0.543 | 0.538 | 0.523 |
| Youden | 1.338 | 1.298 | 1.336 | 1.345 | 1.341 | 1.323 |
| Accuracy | 0.675 | 0.681 | 0.685 | 0.692 | 0.688 | 0.677 |
| Threshold | 0.357 | 0.328 | 0.339 | 0.346 | 0.333 | 0.314 |
| **Abbreviations:** AUC, Area under the curve; CI, Confident interval; NPV, Negative predictive value; PPV, Positive predictive value; SVC, Support Vector Machine. | | | | | | |

| **Supplementary Table S6**. Risk stratification based on the threshold based on the optimal model (Gradient Boosting Machine) in the internal and external validation group. | | | | |
| --- | --- | --- | --- | --- |
| Group | Patients | Probability | | P value |
|  |  | Predicted | Observed |  |
| Internal validation | (n=3767) |  |  |  |
| Low risk (≦46.6%) | 1849 | 27.87% | 27.96% (517/1849) | <0.001 |
| High risk (>46.6%) | 1918 | 63.06% | 63.14% (1211/1918) |  |
| External validation | (n=26285) |  |  |  |
| Low risk (≦46.6%) | 19872 | 27.92% | 25.15% (4998/19872) | <0.001 |
| High risk (>46.6%) | 6413 | 61.11% | 61.89% (3969/6413) |  |
