# Supplementary material for: Prediction of Medical Disputes Between Health Care Workers and Patients in Terms of Hospital Legal Construction Using Machine Learning Techniques: Externally Validated Cross-Sectional Study
Source: J Med Internet Res. 2023 Aug 17;25:e46854. doi: 10.2196/46854 (PMC10472173; doi:10.2196/46854)
Supplement: Multimedia Appendix 3 [file jmir_v25i1e46854_app3.docx]

**Supplementary Figures**


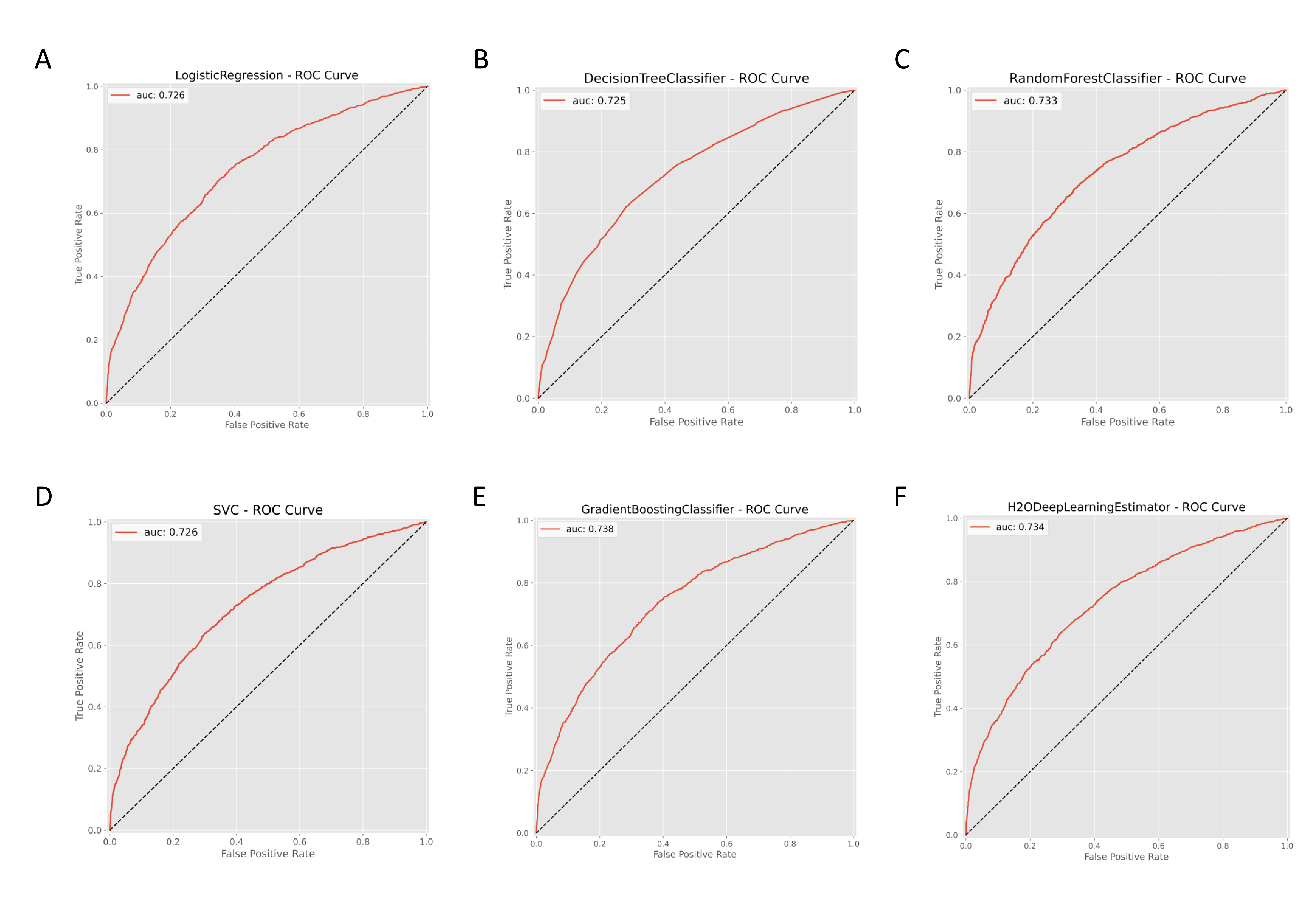


**Supplementary Figure S1.** Area under the receiver operating curve for the six machine-learning models in the internal validation set. A. Logistic Regression; B. Decision Tree; C. Random Forest; D. Support Vector Machine; E. Gradient Boosting Decision Tree; F. Deep Neural Network. The x-axis is false positive rate, and the y-axis is true positive rate. The dotted black line is random prediction.


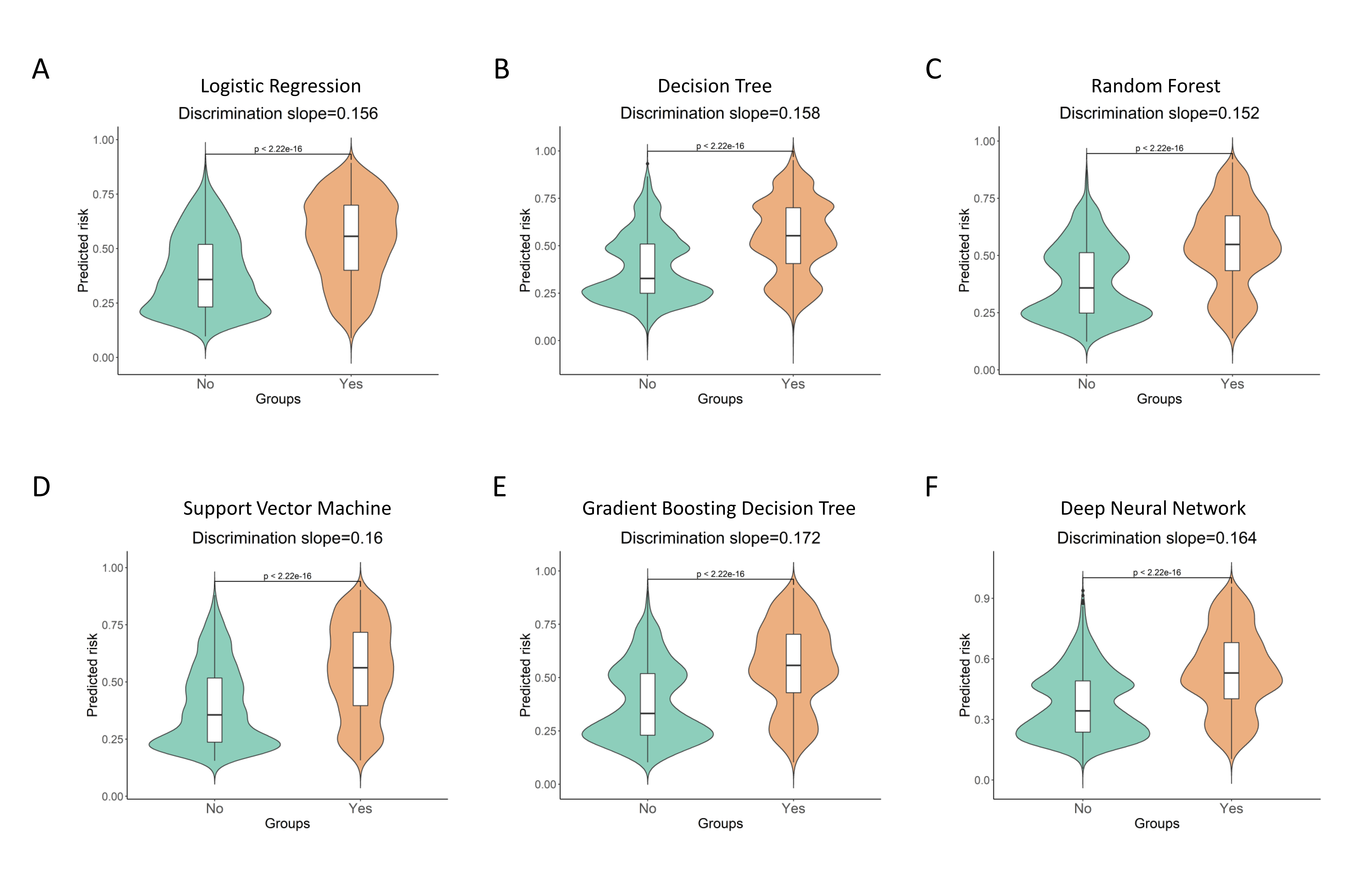


**Supplementary Figure S2.** Violin plots for the discrimination slope of the six machine-learning models in the internal validation set. A. Logistic Regression; B. Decision Tree; C. Random Forest; D. Support Vector Machine; E. Gradient Boosting Decision Tree; F. Deep Neural Network. The discrimination slope is calculated as the difference between the mean predicted probability with and without medical disputes.


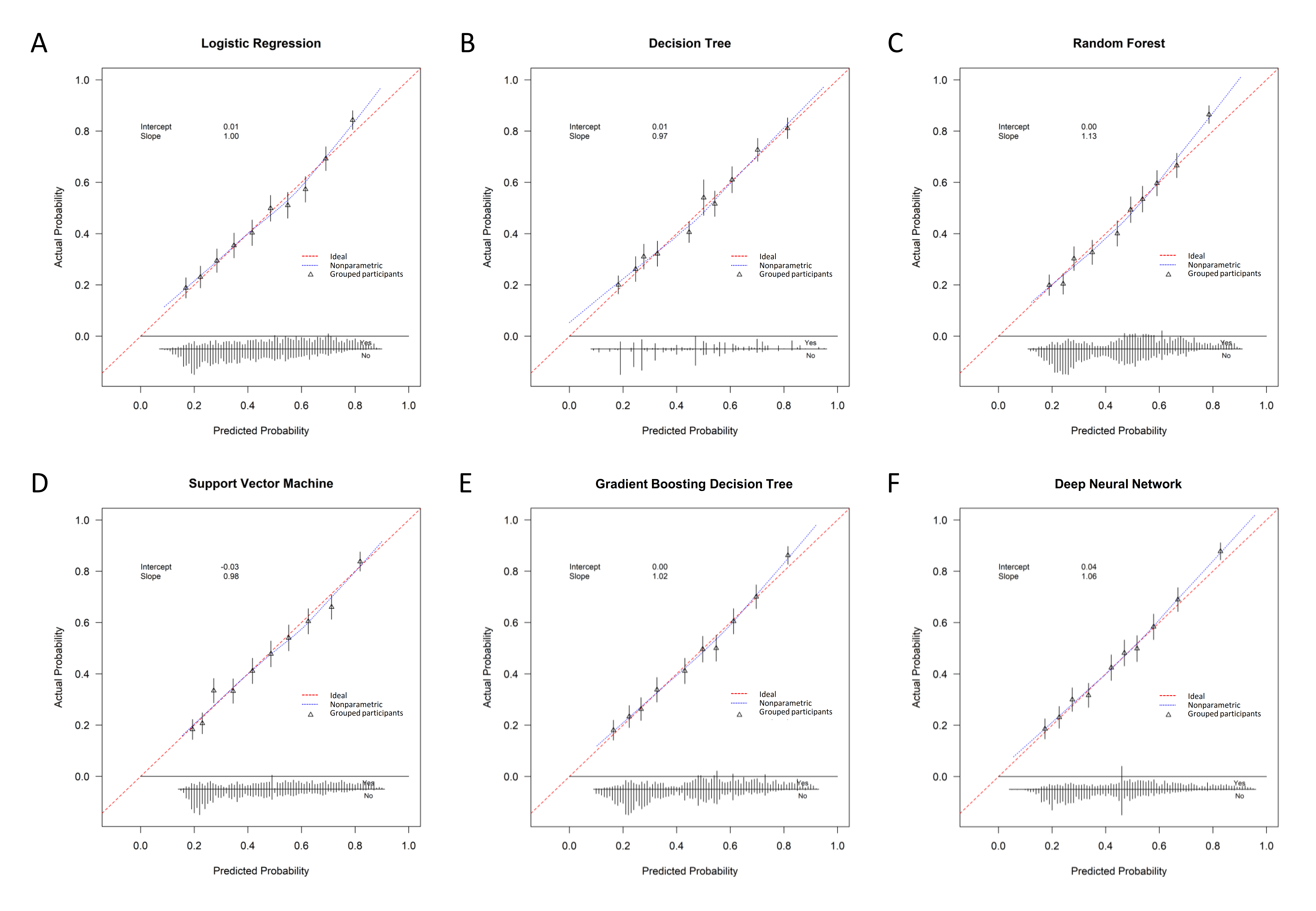


**Supplementary Figure S3.** Calibration curves for the six machine-learning models in the internal validation set. A. Logistic Regression; B. Decision Tree; C. Random Forest; D. Support Vector Machine; E. Gradient Boosting Decision Tree; F. Deep Neural Network. The x-axis is predicted probability, and the y-axis is actual probability. The dotted red line indicates perfect match of models. The closer the dotted blue line is to dotted red line, the better prediction performance the model has.


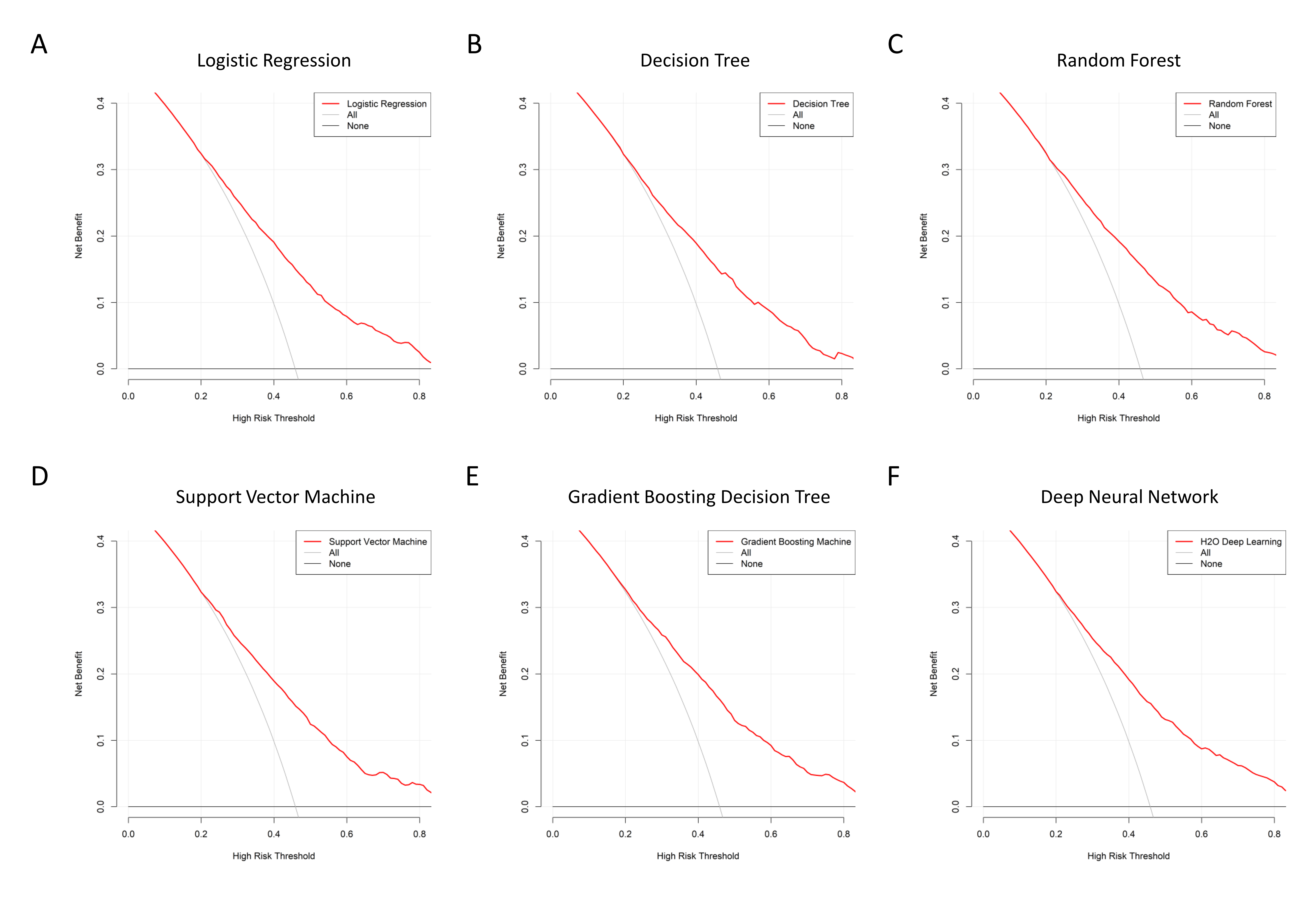


**Supplementary Figure S4.** Decision curve analysis for the six machine-learning models in the internal validation set. A. Logistic Regression; B. Decision Tree; C. Random Forest; D. Support Vector Machine; E. Gradient Boosting Decision Tree; F. Deep Neural Network. The x-ais denotes the risk threshold, and the y-axis indicates the net benefit. The grey line denotes that all patients exhibited early death, and the black line indicates that no patients exhibited medical disputes.


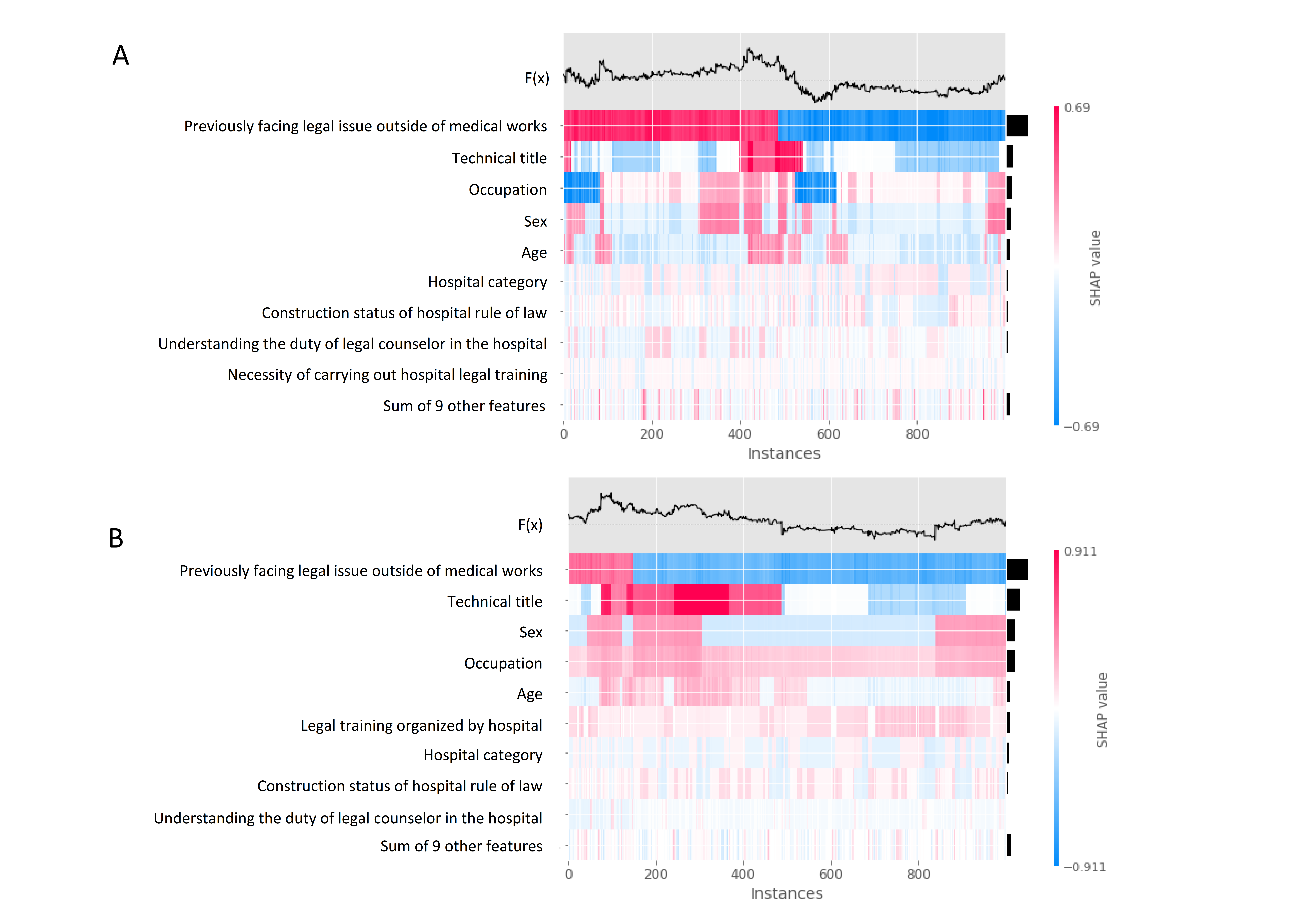


**Supplementary Figure S5.** The heatmap of SHAP value in the first 1000 participants. A. The internal validation; B. The external validation. Features were ranked based on their importance to the outcome.


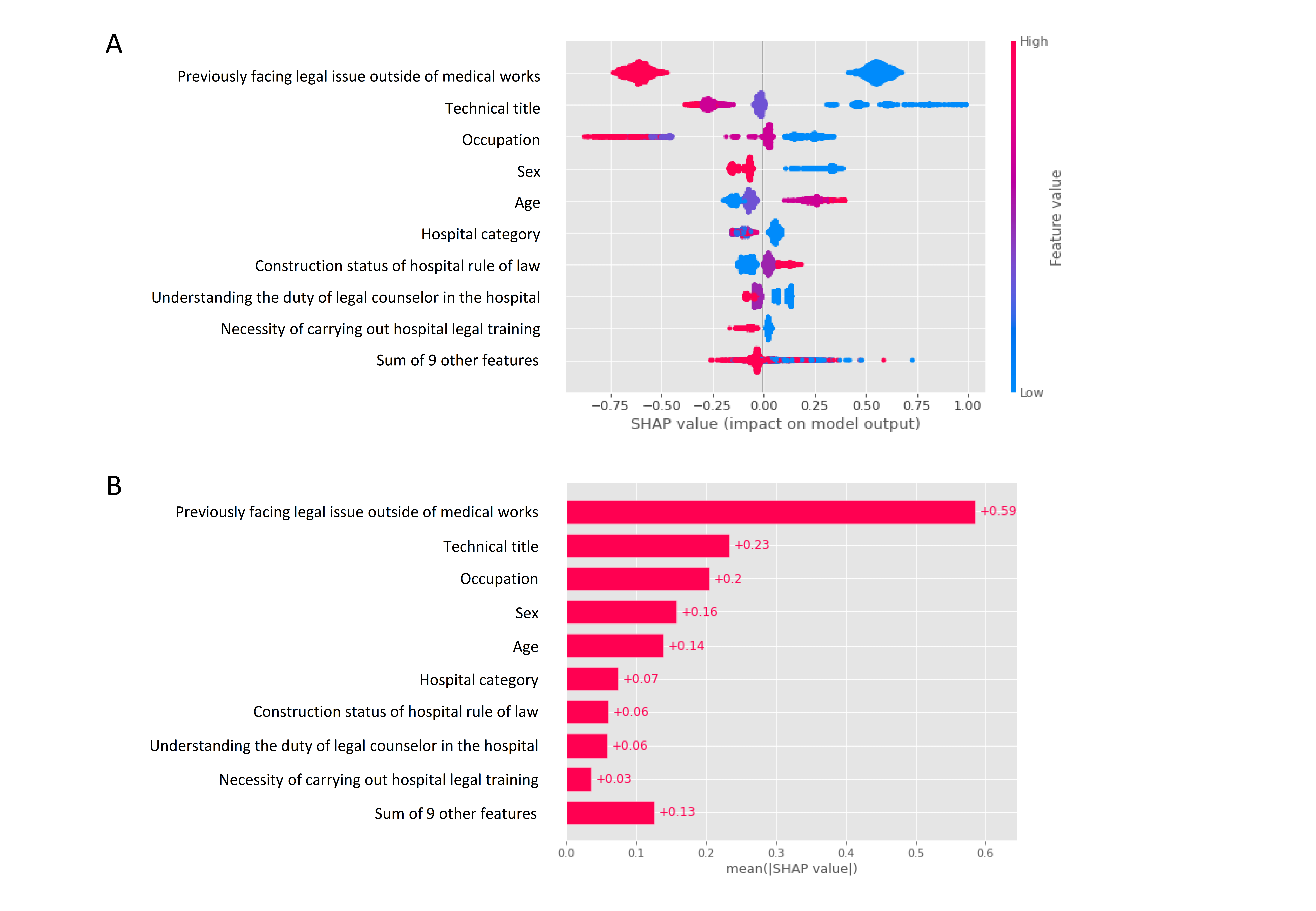


**Supplementary Figure S6.** Analysis of feature importance based on SHAP summary plot in the internal validation set. A. Beeswarm plot of feature importance; B. Bar plot of feature importance.


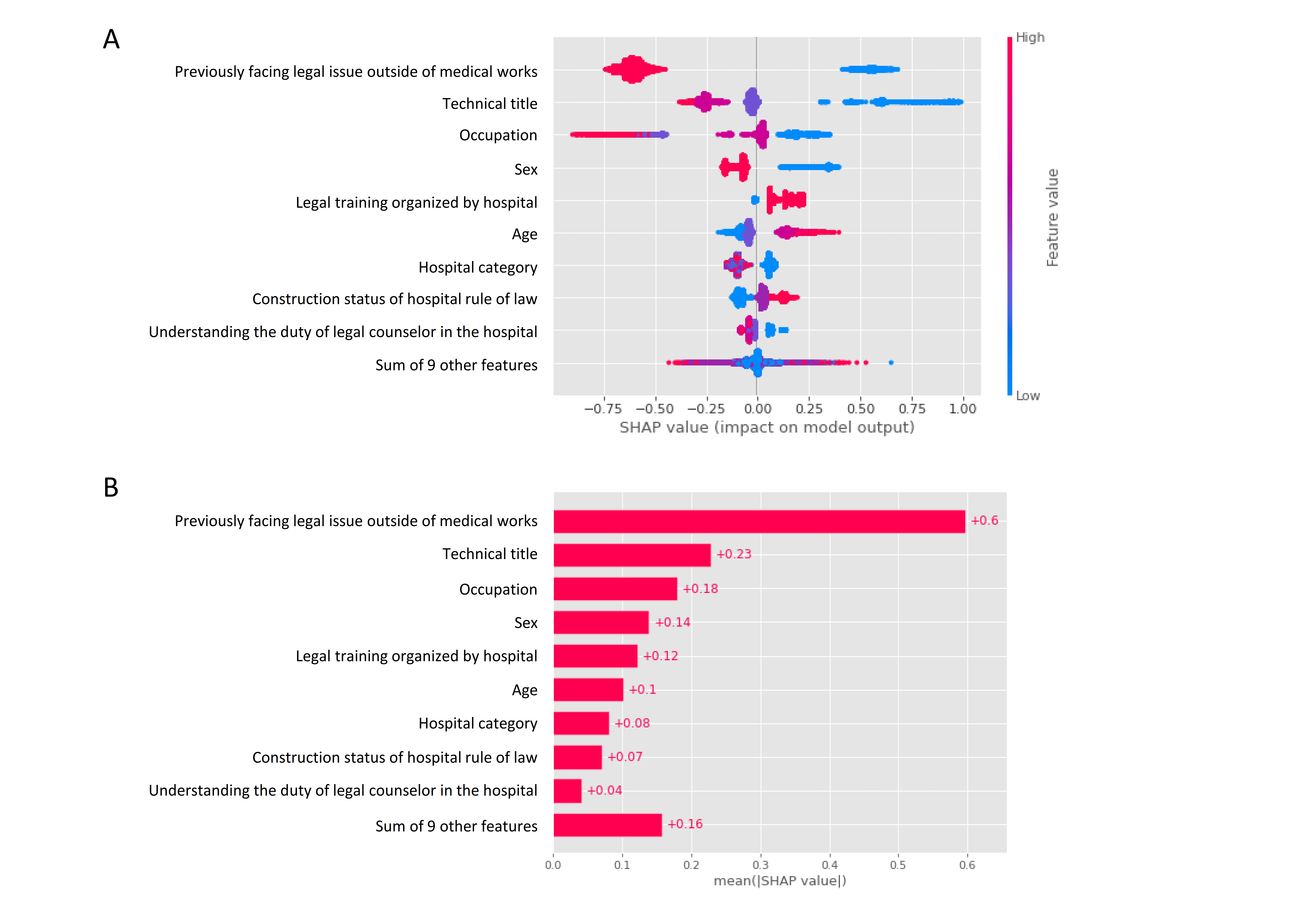


**Supplementary Figure S7.** Analysis of feature importance based on SHAP summary plot in the external validation set. A. Beeswarm plot of feature importance; B. Bar plot of feature importance.


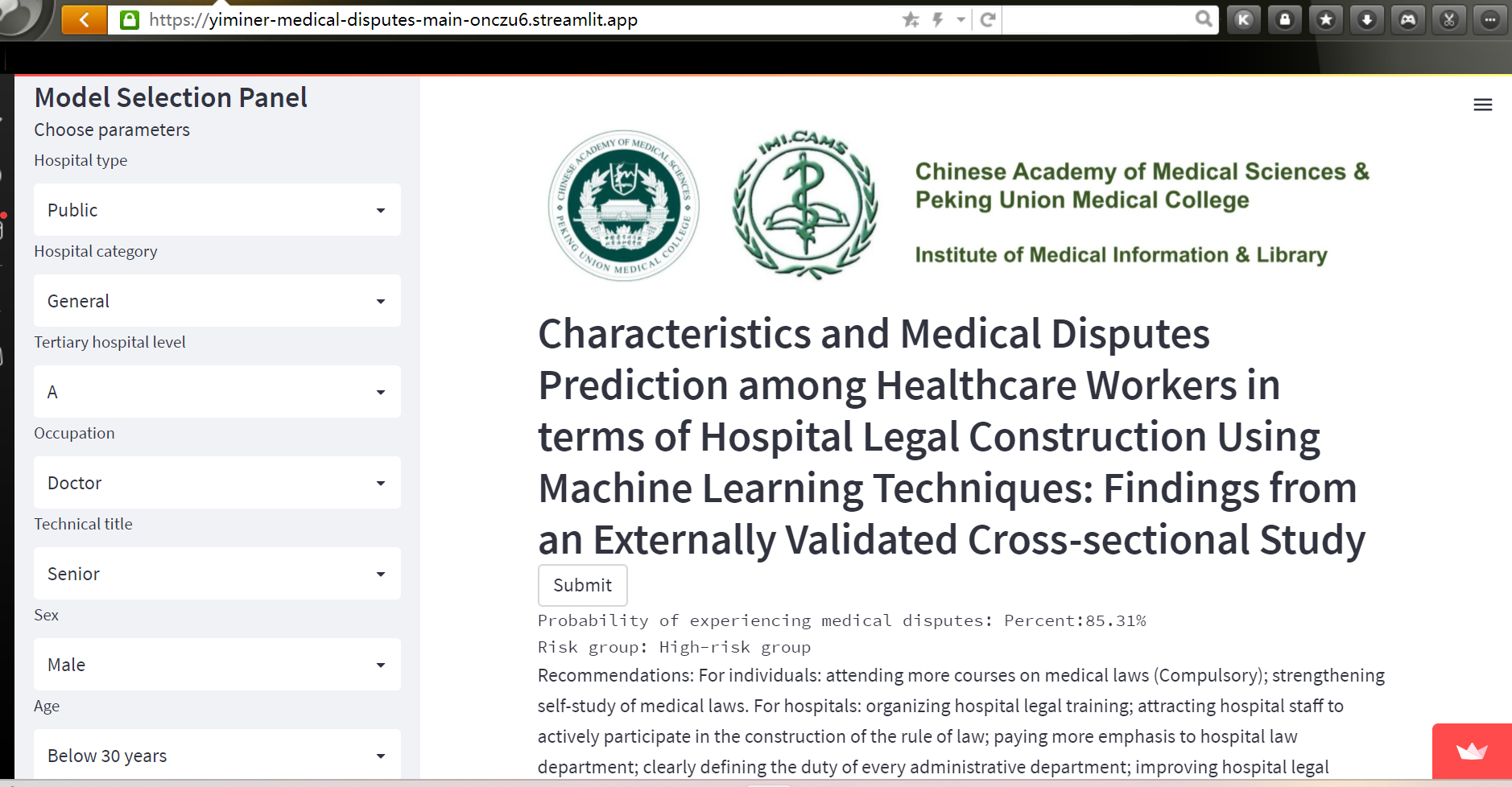


**Supplementary Figure S8.** The web-based application.
